# Functions for rice RFL in vegetative axillary meristem specification and outgrowth

**DOI:** 10.1093/jxb/erv092

**Published:** 2015-03-18

**Authors:** Gauravi M. Deshpande, Kavitha Ramakrishna, Grace L. Chongloi, Usha Vijayraghavan

**Affiliations:** Department of Microbiology and Cell Biology, Indian Institute of Science, Bangalore 560012, India

**Keywords:** Auxin transport, axillary meristem specification, *CUC1*, *CUC2*, *LAX1*, *Oryza sativa*, *RFL*.

## Abstract

Axillary meristems (AMs) are secondary shoot meristems whose outgrowth determines plant architecture. In rice, AMs form tillers, and tillering mutants reveal an interplay between transcription factors and the phytohormones auxin and strigolactone as some factors that underpin this developmental process. Previous studies showed that knockdown of the transcription factor gene *RFL* reduced tillering and caused a very large decrease in panicle branching. Here, the relationship between *RFL*, AM initiation, and outgrowth was examined. We show that *RFL* promotes AM specification through its effects on *LAX1* and *CUC* genes, as their expression was modulated on *RFL* knockdown, on induction of RFL:GR fusion protein, and by a repressive RFL–EAR fusion protein. Further, we report reduced expression of auxin transporter genes *OsPIN1* and *OsPIN3* in the culm of *RFL* knockdown transgenic plants. Additionally, subtle change in the spatial pattern of *IR4 DR5:GFP* auxin reporter was observed, which hints at compromised auxin transport on *RFL* knockdown. The relationship between *RFL*, strigolactone signalling, and bud outgrowth was studied by transcript analyses and by the tillering phenotype of transgenic plants knocked down for both *RFL* and *D3*. These data suggest indirect *RFL*–strigolactone links that may affect tillering. Further, we show expression modulation of the auxin transporter gene *OsPIN3* upon RFL:GR protein induction and by the repressive RFL–EAR protein. These modified forms of *RFL* had only indirect effects on *OsPIN1*. Together, we have found that *RFL* regulates the *LAX1* and *CUC* genes during AM specification, and positively influences the outgrowth of AMs though its effects on auxin transport.

## Highlight

Roles for the transcription factor RFL in rice axillary meristem development were studied. Its regulatory effects on *LAX1*, *CUC1*, and *OsPIN3* reveal its functions in axillary meristem specification and outgrowth.

## Introduction

The aerial architecture of plants is determined by the activity of the shoot apical meristem (SAM) and axillary meristems (AMs) formed in the axils of lateral primordia. The outgrowth of vegetative AMs as shoots branch reiterates the primary shoot developmental pattern and thus gives the plant a bushy architecture. The activity of AMs is dynamically influenced by genetic and environmental factors. In rice, vegetative AMs give rise to tillers and this process involves two key steps: first, the initiation of AMs at leaf axils of juvenile plants that have compact nodes and very limited, if any, internode elongation; and second, the regulated outgrowth of these buds as tillers, a process coincident with internode elongation that occurs along with the transition of the apical meristem to flowering.

Studies on rice mutants with altered tillering have identified a number of genes important for AM initiation and/or the outgrowth of tiller buds (Pautler *et al.*, 2013; Liang *et al.*, 2014). The *moc1* (*monoculm1*) mutant produces a main culm with a limited number of, or no, side tillers. In these plants, flowering transition is normal but panicles have a reduced number of rachis branches and spikelets (Li *et al.*, 2003). The orthologues of *MOC1* are tomato *LATERAL SUPPRESSOR* (*LS*) and *Arabidopsis LATERAL SUPPRESSOR* (*LAS*), and these all encode a member of the GRAS family of plant-specific transcriptional regulators (Li *et al.*, 2003). Rice *LAX PANICLE1* (*LAX1*) encodes a basic helix-loop-helix (*bHLH*) transcription factor, and mutants in this affect AM development. Meristematic cells are specified in *lax1* mutants but they fail to fully progress to form AMs (Oikawa and Kyozuka, 2009).

The outgrowth of the axillary buds is influenced by hormonal signals and by environmental cues like plant density, nutrient availability, and light. Studies on *more axillary growth* (*max*) *Arabidopsis* branching mutants, the *dwarf* (*d*) group of rice tillering mutants, and *ramosus* (*rms*) mutants in pea identified the inhibitory role of strigolactones (Stirnberg *et al.*, 2002; Gomez-Roldan *et al.*, 2008; Umehara *et al.*, 2008). Amongst the genes involved in this pathway, *MAX3*/*D17*/*HIGH TILLERING DWARF1* and *MAX4*/*D10* encode biosynthetic enzymes (Zou *et al.*, 2006; Arite *et al.*, 2007), while *MAX1*, a *CYPTOCHROME P450* (*CYP*) superfamily member of the CYP711 clade, is involved in later stages of strigolactone biosynthesis (Booker *et al.*, 2005). The *MAX2*/*D3* gene encodes a protein member of the F-box family that forms the substrate recognition subunit of the Skp, Cullin, and F-box (SCF) ubiquitin E3 ligases that mediate regulated protein degradation. In rice, some additional genes, *D14*, *D27* and *D53*, are also known that encode factors involved in strigolactone biosynthesis, protein–protein interactions, or signalling (Arite *et al.*, 2009; Lin *et al.*, 2009; Jiang *et al.*, 2013).

Phytohormones that influence the outgrowth of AMs are auxin and cytokinin. Auxin synthesized at the growing tip of the plant is transported basipetally by the polar auxin transport stream (PATS). The direction of auxin movement through tissues is determined by the asymmetric cellular localization of members of the *PINFORMED* (*PIN*) family of transporters (Wisniewska *et al.*, 2006). Further, at the cellular level, *PIN* genes are regulated by transcriptional control and the dynamic subcellular localization of PIN proteins. The serine/threonine protein kinase PINOID (PID) regulates PIN localization. The rice counterparts OsPIN1 and OsPID are key factors for polar auxin transport (Xu *et al.*, 2005). Recent studies in *Arabidopsis* elucidate some mechanistic details underlying auxin, apical dominance, and shoot branching. One model proposes that strigolactones act downstream of auxin and directly inhibit axillary bud outgrowth, possibly by regulating meristem activity (Brewer *et al.*, 2009; Brewer *et al.*, 2013). Other studies with *Arabidopsis max* mutants show that strigolactones may control the outgrowth of axillary buds by modulating polar auxin transport itself. These studies propose that, depending on polar auxin transport status, strigolactones may mediate the release of auxin from buds into the stem and thereby determine the number of buds to be activated (Crawford *et al.*, 2010; Shinohara *et al.*, 2013).

The rice *LEAFY* orthologue *RICE FLORICULA/LEAFY* (*RFL*), also called *ABERRANT PANICLE ORGANIZATION 2* (*APO2*), is expressed at high levels in very young branching panicles (Kyozuka *et al.*, 1998; Prasad *et al.*, 2003); before this, in juvenile vegetative plants, *RFL* is expressed in the axils of leaves (Rao *et al.*, 2008). The latter expression pattern of *RFL* and the reduced tillering phenotype seen on knockdown of *RFL* (Rao *et al.*, 2008) prompted this study. Here, we probed the relationship between *RFL* and other factors that control AM development. The expression status of key transcription factor genes, auxin transporters and components in the strigolactone pathway gave an insight on the interplay between *RFL* and some of these key downstream factors. Our data suggest that *RFL* could prompt tiller specification and outgrowth by its cumulative effects on meristematic genes such as *LAX1* and *CUP SHAPED COTYLEDON1* (*CUC1*), and effects on auxin transport by regulation of *OsPIN3*.

## Materials and methods

### Plant materials

Rice (*Oryza sativa* L. var. japonica) TP309 embryogenic calli were used to raise transgenic plants via *Agrobacterium-*mediated transformation as described by Prasad *et al.* (2001).

### RT-PCR and real-time PCR

For expression analysis in *dsRNAiRFL*, *UbiRFL*(*as*)*-1*, *pUGN RFL:GR* or *I2B:RFL-EAR* transgenic plants, RNA was isolated by the Trizol method (TRI reagent, Sigma) as per the manufacturer’s instructions. 2 μg of total RNA was used for cDNA synthesis using MMLV reverse transcriptase (Fermentas) and oligo dT20 as the reverse primer. 25ng cDNA was used for each qPCR with 5 µM gene-specific primers and Sensimix SYBR (Bioline). For calculating the fold change in the expression of genes in *dsRNAiRFL*, *UbiRFL*(*as*)*-1*,*UGN RFL:GR* or *I2B:RFL-EAR* axillary tissues, transcript levels in wild-type and transgenic axillary tissues were first normalized to the levels of actin, taken as the normalizing control, to obtain Ct values. The 2-^ΔΔCt^ values were then calculated to determine the fold change of expression as described by Yadav *et al.* (2007), with the standard error calculated from six replicates derived from two biological samples. Primers used are listed in Supplementary Table S1. For validating the transgenic status of *dsRNAiRFL* or *Ubi amiR RFL* transgenic plants, RNA was isolated by the Trizol method (TRI reagent, Sigma) as per the manufacturer’s instructions from leaf tissues and reverse transcription reactions were performed. Wild-type tissues were used as negative controls. 50ng cDNA was used as a template for PCR reactions with transcript-specific primers.

### Histological analysis and microscopy

Rice culms containing SAM with leaf sheaths were fixed in formalaldehyde/acetic acid/alcohol, stained with 1% eosin in 90% alcohol, and dehydrated through a graded ethanol series. The tissues were then embedded in Paraplast Plus (Sigma) and 8 µM sections prepared for bright-field microscopy (Zeiss Axioskop2, Germany). Images of the vegetative shoot in control *IR4 DR5:GFP* and *dsRNAiRFL-IR4DR5:GFP* knockdown plants (~50 days old) were captured on a Zeiss LSM710 laser scanning confocal microscope using a 20× Air Objective (NA 0.8). Signals were collected using a 488nm laser line with 5% power and the pinhole set to 2 Airy Unit (Ex/Em of 493nm/524nm). Images were further processed by ImageJ software (http://imagej.nih.gov/ij/, accessed 26 February 2015) and assembled in Adobe Photoshop.

### 1-*N*-naphthylphthalamic acid treatment

For 1-*N*-naphthylphthalamic acid (NPA) treatment, wild-type and *UbiRFL*(*as*)*-1* seeds were sterilized with 70% ethanol followed by 40% bleach, and were then washed thoroughly with sterile water. These seeds were then inoculated on plates using 1/2MS media with varying concentration of NPA (Sigma). The plates were incubated vertically during seedling growth for 10 days in 16h/8h light/dark cycles. The seminal root length was determined for five plants taken for each treatment.

### Dexamethasone-based induction of RFL∆GR

15-day-old T1 seedlings of *pUGNRFL:GR-2* and control wild-type seedlings of similar age were treated with 10 µM dexamethasone or 0.1% ethanol as a mock control. Five plants per treatment were grouped to form two biological replicates and the treatment regime was for 9h. RNA was prepared from culm tissues of these groups of plants (wild type and *pUGNRFL:GR-2*) by the Trizol method (TRI reagent, Sigma) as per the manufacturer’s instructions.

## Results

### AM development in RFL knockdown plants

**Fig. 1.**
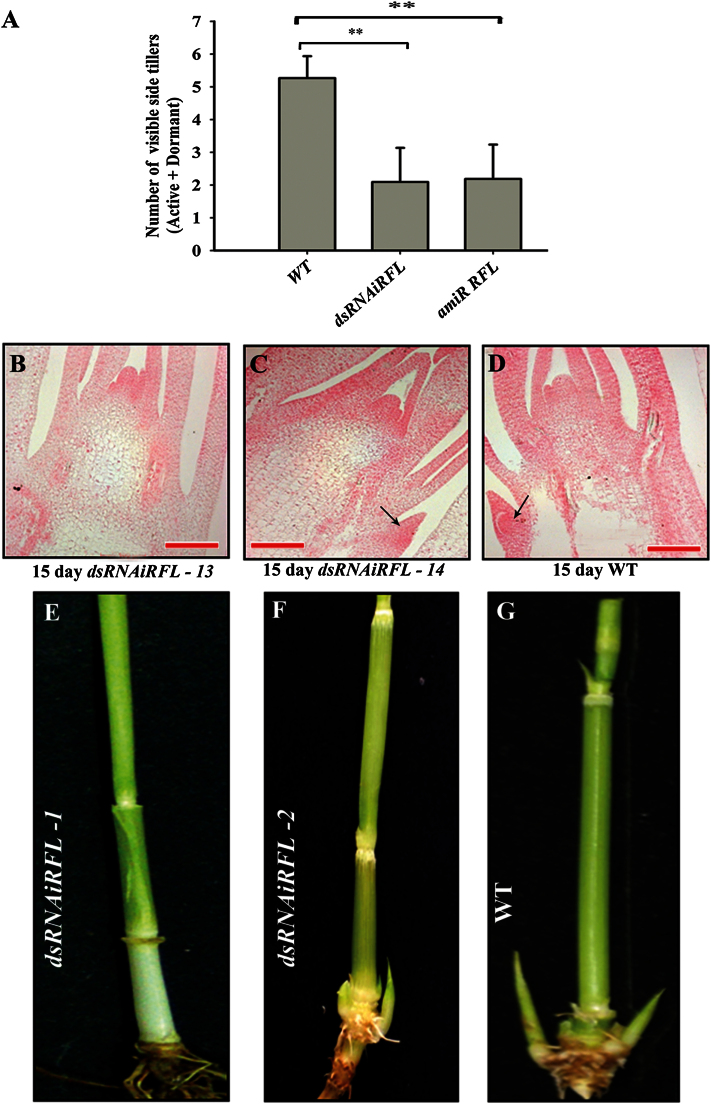
Plants with *RFL* knockdown show a defect in AM specification and outgrowth. (A) Number of visible tillers in *dsRNAiRFL*, *amiR RFL* and wild-type (WT) flowering plants with 0.2cm panicles (*n* = 12 for *dsRNAiRFL*; *n* = 16 for wild type and *amiR RFL*). The error bars represent the standard error of the mean. (B–D) Eosin-stained longitudinal sections passing through the shoot apical meristem of hardened 15-day-old plants. The status of AMs in *dsRNAiRFL-13* (B), *dsRNAiRFL-14* (C), and control wild type (D) are shown with black arrows indicating axillary buds. Scale bars: 100 µm. (E–F) Representative display of AM status in plants after their transition to flowering: *dsRNAiRFL-1* (E); *dsRNAiRFL-2* (F); and a tissue culture-regenerated wild-type plant (G). The leaves and leaf sheaths were removed to show the tiller buds. **, *P* < 0.01.

To examine tillering defects on knockdown of *RFL*, a binary construct *dsRNAiRFL* expressing hairpin RNAs against *RFL* was used (Rao *et al.*, 2008). We generated 14 independent transgenic lines and 14 control wild-type regenerated plants were raised in parallel. Leaf tissues in *dsRNAiRFL* transgenics were used to confirm their transgenic status by testing for the presence of hairpin RNAs against *RFL*. All 14 lines were positive (Supplementary Figure S1) and hence were taken for phenotypic analysis. The status of AMs in two of these plants (*dsRNAiRFL-13* and *dsRNAiRFL-14*) was examined in eosin-stained sections of the vegetative culm in 15-day-old hardened plants. As compared to a culm section of a tissue culture-regenerated wild-type plant of similar age, the culm in *dsRNAiRFL-13* had not initiated any AMs (Fig. 1B, compare with the wild type in 1D). In the *dsRNAiRFL-14* line, buds were greatly reduced in number and their outgrowth was compromised (Fig. 1C). The remaining 12 lines were allowed to develop further until flowering transition of their shoot apical meristem had occurred and a young panicle of 0.2cm had formed. The time taken for this developmental transition was delayed in the *RNAi* lines (Supplementary Figure S1), as is expected for loss-of-*RFL*-function plants (Rao *et al.*, 2008; Ikeda-Kawakatsu *et al.*, 2012). Further, a lower number of visible axillary buds were noted as compared to the bud number in tissue culture-regenerated wild-type plants (Fig. 1A). At an age when apical meristems are converted to ~0.2cm panicles, six *dsRNAiRFL* lines (*dsRNAiRFL-1*, *dsRNAiRFL-3*, *dsRNAiRFL-4*, *dsRNAiRFL-8*, *dsRNAiRFL-10*, and *dsRNAiRFL-12*) displayed defects in AM specification and outgrowth (Fig. 1E). Six other lines (*dsRNAiRFL-2*, *dsRNAiRFL-5*, *dsRNAiRFL-6*, *dsRNAiRFL-7*, *dsRNAiRFL-9*, and *dsRNAiRFL-11*) had poor growth of axillary buds (Fig. 1F). These data were compared to bud numbers in 14 tissue culture-regenerated wild-type plants of similar developmental age (Fig. 1A and G). We also generated a knockdown of *RFL* by the ubiquitous expression of an artificial microRNA. Sixteen of these *amiR-RFL* lines, confirmed for the presence of the transgenic T-DNA, were studied phenotypically (Supplementary Figure S1). These lines displayed delayed transition to flowering as they took ~100 days as opposed to ~64 days in tissue culture-regenerated wild-type plants (Supplementary Figure S1). Also, the *amiR-RFL* plants had fewer visible tillers than tissue culture-regenerated wild-type plants (Fig. 1A).

### Reduction of RFL activity alters expression status of AM development genes

**Fig. 2.**
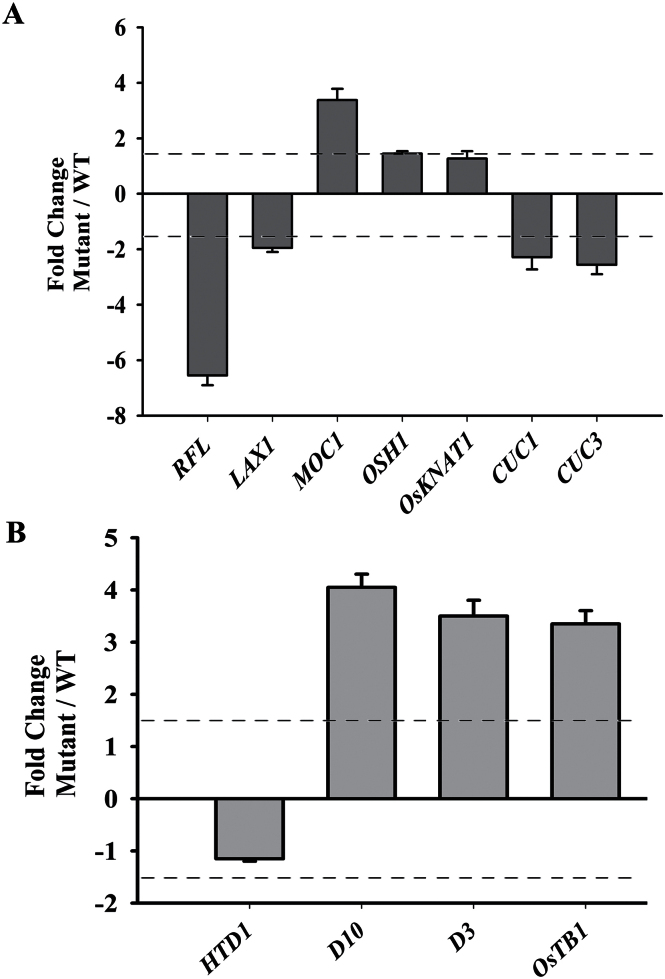
Expression status of genes involved in AM development is influenced by *RFL*. (A) RT-qPCR analyses of transcripts from candidate AM regulatory genes in AM tissues. The normalized transcript levels in *dsRNAiRFL* plants were compared to the wild type (WT). (B) RT-qPCR analyses of expression levels of strigolactone pathway genes in AM tissues from *dsRNAiRFL* and wild-type plants. For all genes the fold change in normalized expression levels is represented as the mean with standard error of the mean (*n* = 6). A fold change of 1.5 was taken to categorize genes regulated by *RFL* and is indicated by the dotted line in the graph.

To explore the relationship between the expression status of *RFL* and pathways known to support AM specification and outgrowth, transcript levels for some other key transcription factors and signalling pathways involved in AM development were examined. Axillary meristem tissues were dissected from eight transgenic lines of *dsRNAiRFL* knockdown plants and used to generate two biological RNA pools. Similar numbers of control tissue culture-regenerated wild-type plants were dissected in parallel to generate control AM RNA samples. In culm tissues from knockdown plants, we noted about 7-fold *RFL* downregulation as compared to transcript levels in wild-type tissues (Fig. 2A). LAX PANICLE1 (LAX1), a bHLH transcription factor and orthologue of maize BARRENSTALK1 (BA1), is expressed in cells at the margins between the shoot apical meristem and peripheral regions from where new primordia emerge. The *lax1* mutants fail to specify AMs (Komatsu *et al.*, 2003). We observed a 2-fold downregulation of *LAX1* transcripts in the culm of *RFL RNAi* plants (Fig. 2A). These results support the anatomical analyses of *RFL* knockdown plants presented earlier, which showed reduced or no buds in these transgenics. Transcript levels for rice *MOC1*, which plays an important role in the initiation of AMs, was increased about 3-fold as compared to the levels in wild-type culm tissues. The rice homeobox gene *ORYZA SATIVA HOMEOBOX 1* (*OSH1*) serves as a molecular marker for meristematic cells and it is the closest homologue of *Arabidopsis STM1.* Expression of *OSH1* in rice AMs precedes the expression of *LAX1* (Oikawa and Kyozuka, 2009). In *RFL* knockdown plants, *OSH1* transcript levels were unaffected in the culm tissues (Fig. 2A). Another homeobox gene, *OsKNAT1* (*OSH15*), normally expressed in leaf axils (Sentoku *et al.*, 1999), was also unaffected (Fig. 2A). The CUC1, CUC2, and CUC3 proteins, which belong to the NAC domain family, function redundantly to establish boundaries between SAM and emerging primordia (Aida *et al.*, 1997; Vroemen *et al.*, 2003). More recently, functions for *CUC3* in AM initiation have been reported in *Arabidopsis* (Raman *et al.*, 2008). Hence, we examined the expression status of these genes in rice culms from wild-type and *RFL* knockdown plants. As *CUC1* and *CUC2* share significant functional overlap, and are both regulated by miR164, a set of primers to detect their combined levels was used, while *CUC3* was measured independently. We found reduced transcript levels for both *CUC1* and *CUC3* genes in *RFL* knockdown tissues (Fig. 2A).

**Fig. 3.**
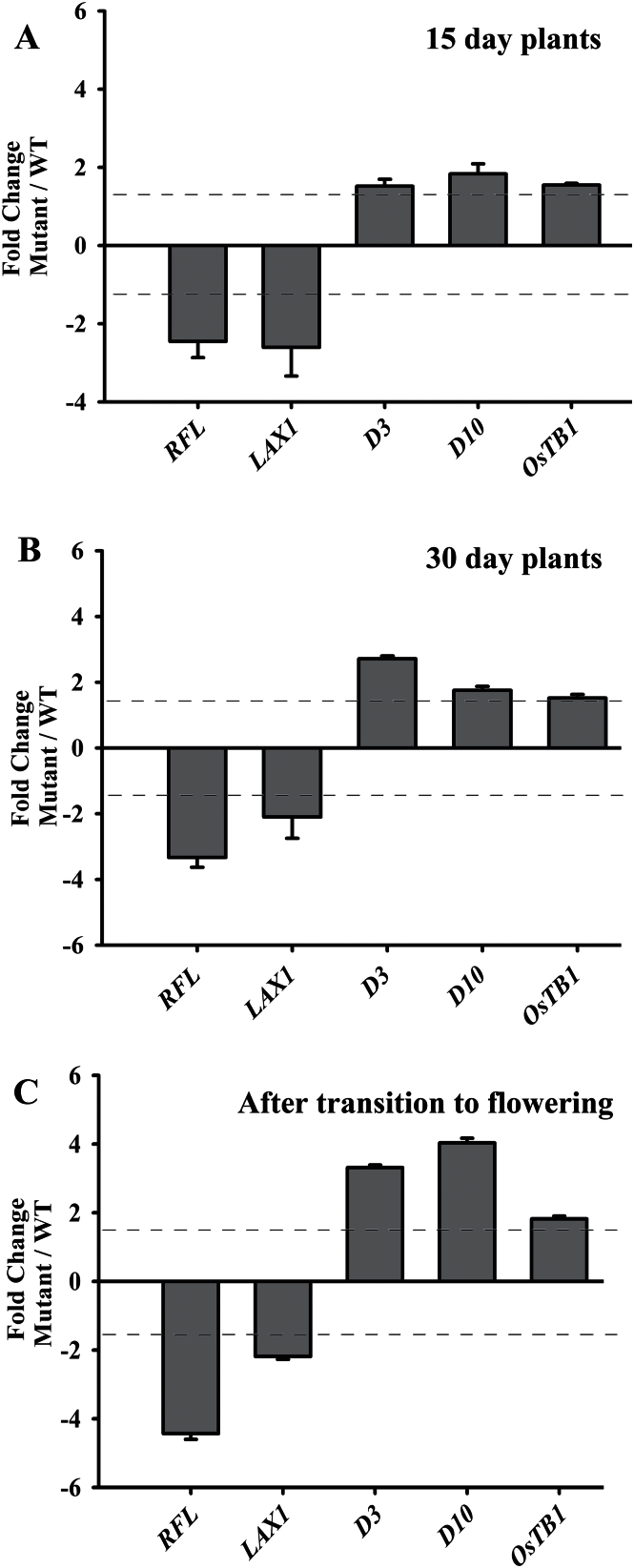
Expression analyses of some AM-pathway genes in plants at different developmental growth stages. RT-qPCR analyses of some AM pathway genes in culm tissues of plants with partial knockdown of *RFL* and in wild-type (WT) plants. The fold change in transcript levels is shown in 15-day-old *UbiRFL* (*as*) compared to wild type (A), in 30-day-old plants (B), and in plants after their transition to flowering (C). In each case, the fold change in normalized expression levels is represented as the mean with standard error of the mean (*n* = 6). A fold change of 1.5 was taken to categorize genes regulated by *RFL* and is indicated by the dotted line in the graph.

After the specification of AMs, their outgrowth is influenced by strigolactone and auxin-signalling pathways. We investigated the expression status of strigolactone biogenesis genes *HTD1* and *D10*, encoding CAROTENOID CLEAVAGE DIOXYGENASE 7 and 8, respectively. We also determined transcript levels for *D3*, encoding F-box/LRR-repeat protein involved in hormone perception. The transcript levels of *HTD1* were not significantly altered in *RFL* knockdown tissues (changes greater than 1.5-fold were taken as significant). However, nearly 4-fold upregulation in transcript levels of *D10* and *D3* genes occurred in the culm of *RFL* knockdown plants as compared to wild-type tissues (Fig. 2B). To understand if the effects of *RFL* on expression status of these genes are discernible in plants at earlier developmental stages, we studied culm tissues from juvenile plants for transcript levels of *LAX1*, *D3*, *D10*, and *OsTB1* genes. For these experiments we utilized seed-germinated transgenics with a partial knockdown of *RFL* achieved through expression of antisense RNA against *RFL* (Rao *et al.*, 2008). We took this approach as strong knockdown of *RFL* leads to much reduced flower number and seed set (Rao *et al.*, 2008; Ikeda-Kawakatsu *et al.*, 2012). Seedlings from the *UbiRFL* (*as*)*-1* line were grown and we dissected the culm both from plants 15 and 30 days post-germination and from plants that had undergone the transition to flowering. Similarly staged wild-type tissues were also collected. With comparative analysis, we found transcript levels of *LAX1* were affected even in 15-day-old *RFL* partial knockdowns, a defect which persisted in 30-day-old seedlings and in the culm of plants that were flowering. On the other hand, changes in expression levels of *D3* and *D10* were obvious in 30-day-old plants and in plants that had made a transition to flowering (Fig. 3A–C). A slight increase in *OsTB1* transcript levels was discernible only in plants that had undergone the transition to flowering (Fig. 3C). Interestingly, *OsTB1* expression is unaffected by strigolactone addition (Minakuchi *et al.*, 2010). These data suggest that *RFL* may influence *OsTB1* by alternative mechanisms. Together, these data on regulators of AM specification and outgrowth indicate an early developmental defect in AM specification with progressive deregulation in strigolactone synthesis and perception in plants that have compromised *RFL* activity.

### Interaction of RFL, strigolactone signalling, and bud outgrowth

**Fig. 4.**
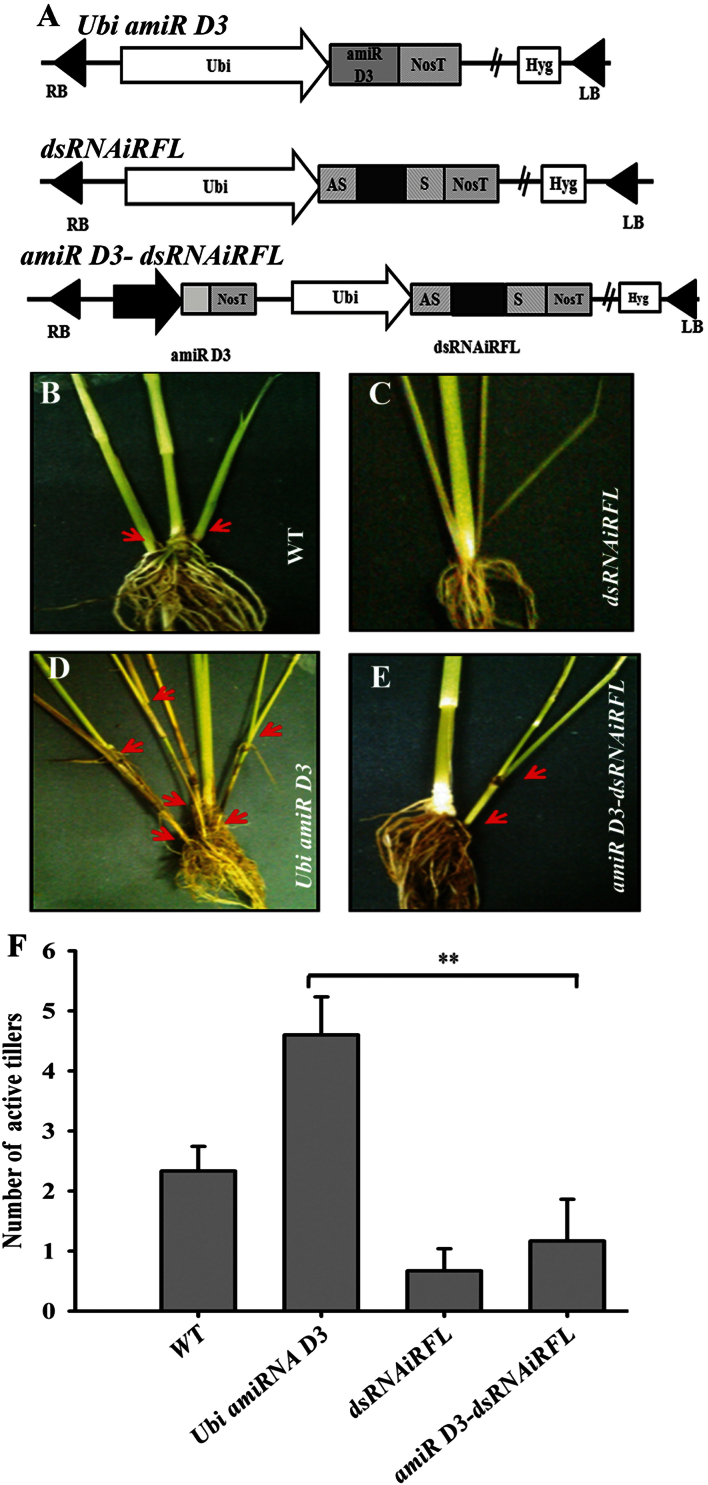
(A) Schematic representation of the T-DNA segment in constructs used for knockdown of *D3* (*Ubi amiRD3*), *RFL* (*dsRNAiRFL*), and *D3* and *RFL* (*amiR D3-dsRNAiRFL*). (B–E) Representative images of culms with active AMs in wild-type (WT) (B), *dsRNAiRFL* (C), *UbiamiR D3* (D), and *amiRD3-dsRNAiRFL* (E) plants. Red arrows indicate AMs. (F) Quantitative representation of active tiller numbers in plants of the given genotypes. Tiller number was counted after emergence of the panicle from the main stem. The error bars indicate the standard error of the mean. *amiRD3-dsRNAiRFL*, *n* = 6; *UbiamiRD3*, *n* = 5; *dsRNAiRFL*, *n* = 6; wild type, *n* = 6. **, *P* < 0.01.

Transcripts from the *D3* gene are expressed in a wide range of tissues including leaves, vegetative culm (with SAM and AMs), roots, young panicles (0.1–0.3cm) and dissected AMs (Supplementary Figure S3). The tissue distribution of *Arabidopsis MAX2* transcripts has been studied using promoter–reporter fusions, and activity was restricted to meristematic and vascular tissues of juvenile and adult plants (Shen *et al.*, 2007). In rice, *RFL* transcripts are spatially and temporally regulated. It is expressed in the vegetative culm and dissected axillary tissues, and its transcripts are dynamically regulated in developing panicles. Transcripts are not detected in mature leaves and roots (Supplementary Figure S3; Kyozuka *et al.*, 1998; Rao *et al.*, 2008). To examine any relationship between *RFL* and the strigolactone pathway, we carried out knockdown of *D3* in plants that were otherwise wild type and in plants that were knocked down for *RFL* (Fig. 4A). Transgenic lines that harboured a single copy of the integrated T-DNA (*dsRNAiRFL*, *Ubi amiR D3*, or *amiR D3-dsRNAiRFL*; Supplementary Figure S4) were analysed phenotypically. Five independent single-copy T-DNA lines with artificial microRNA-driven knockdown of *D3* were obtained. These plants had increased tillering (Fig. 4D) and reduced plant height as compared to the control wild-type regenerated plants (Fig. 4B, Supplementary Figure S5) indicating lowered strigolactone pathway activity. These phenotypes partially mimic, with lesser severity, the effects of a T-DNA insertion at the *D3* locus (Ishikawa *et al.*, 2005). In this context we examined the consequences of *RFL* knockdown. Six independent transgenic lines with single-copy T-DNA expressing amiRNA against *D3* and hairpin RNAs against *RFL* were studied for their tillering phenotype. These plants had an average of only one active tiller, a significant reduction when compared to an average five active tillers in lines with knockdown of only *D3* (Fig. 4E, F). The number of active tillers in these *amiR D3-dsRNAiRFL* plants was comparable but not statistically equivalent to that in *RFL* knockdown plants (Fig. 4F). Thus, *RFL* functions, through other interacting pathways, are important for tiller outgrowth even when strigolactone activity is dampened. To investigate the likelihood of a causal link between strigolactone and auxin transport proteins during tiller outgrowth, we studied *OsPIN1* and *OsPIN3* transcript levels in wild-type plants treated with GR24, a synthetic analogue of strigolactones. We observed that plants of three developmental stages (15 and 30 days, and after flowering transition) treated with GR24 had reduced levels of *OsPIN1* transcripts in vegetative culm tissues, but this had no effect on *OsPIN3* transcript levels (Supplementary Figure S5A and E). Taken together, our data show that strigolactones can influence *OsPIN1* expression status, but the role played by *RFL* in this interaction requires further investigation.

### RFL knockdown plants are compromised for auxin transport

**Fig. 5.**
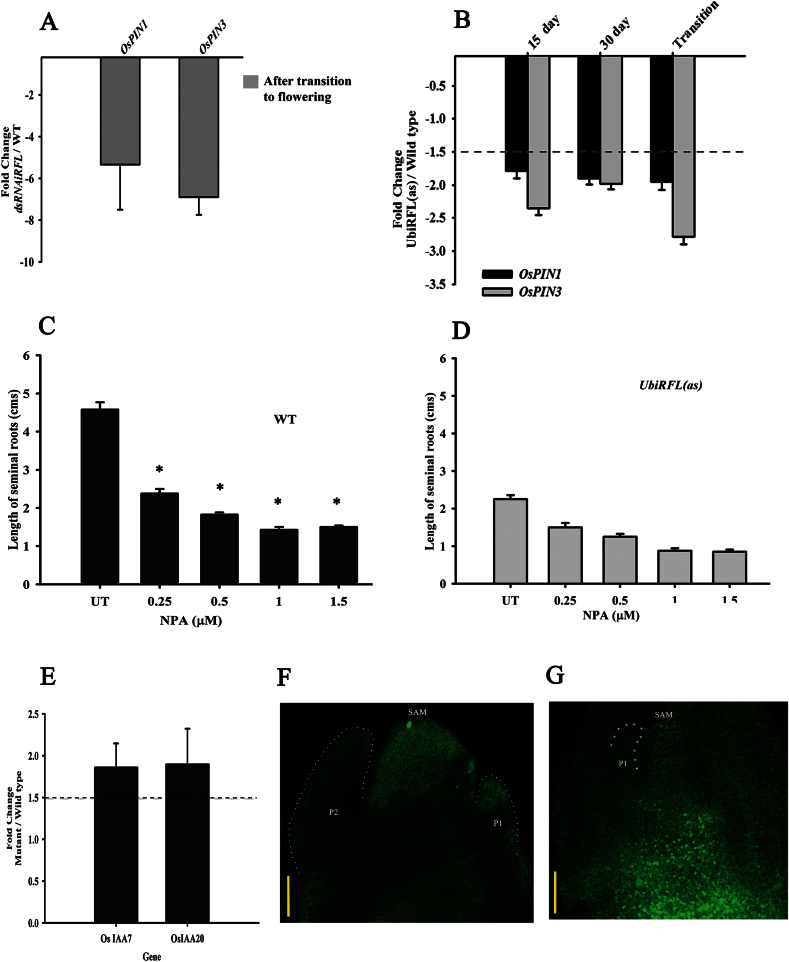
*RFL* knockdown plants have altered auxin transporter gene expression. (A) RT-qPCR of *OsPIN1* and *OsPIN3* transcripts in culm tissues of *dsRNAiRFL* and wild-type (WT) plants with young panicles. The plot shows the mean fold change with standard error of the mean (*n* = 6). (B) Transcript levels for *OsPIN1* and *OsPIN3* in the culm of plants at different developmental stages. Expression level in *UbiRFL* (*as*) plants was compared to the wild type. Plants were either 15 days old, 30 days old, or after transition to flowering. A fold change of 1.5, indicated by a dotted line in the graph, was taken to categorize genes regulated by *RFL*. (C, D) Effects of NPA, a physiological inhibitor of auxin transport, on seminal root length. (C) NPA-treated wild-type seedlings have a significant reduction in root length (*, *P <* 0.01). The mild reduction in root length of *Ubi::RFL* (*as*) seedlings was not statistically significant at *P <* 0.01. UT, untreated. The error bars represent the standard error of the mean (*n* = 10). (E) Transcript levels for *OsIAA7* and *OsIAA20* in the culm of *dsRNAiRFL* and wild-type flowering plants. The mean fold change, with standard error of the mean (*n* = 6), is plotted. (F, G) Auxin reporter distribution in wild-type and *RFL* knockdown plants. A distinctive spatial organization of auxin reporter activity is seen in the vegetative shoot apex of the control plant (F, *IR4DR5-GFP*); in the *RFL* knockdown plant (G, *dsRNAiRFL-IR4DR5:GFP*) dispersed signal is detected in the shoot apex, leaf primordia, and nodal region. Scale bars: 50 µm.

Recent studies indicate that strigolactone regulates bud outgrowth by altering the PATS (Shinohara *et al.*, 2013). The PIN family of auxin efflux carriers plays a predominant role in the PATS. Their intracellular localization determines the direction of auxin flow and is also critical for the positioning and initiation of a variety of lateral meristems and organ primordia (Reinhardt *et al.*, 2000). In panicles of *RFL* knockdown plants our earlier studies on gene expression profiles showed that the auxin efflux facilitator *OsPIN3-*like (AK101504) was downregulated (Rao *et al.*, 2008). Here, we investigated the expression levels of *OsPIN1* and *OsPIN3* in the culm tissues of *RFL* knockdown plants (*dsRNAiRFL*) after their transition to flowering. We detected downregulation of *OsPIN1* expression by about 5-fold and of *OsPIN3* by 7-fold (Fig. 5A). To understand if the effects of *RFL* on expression status of these *PIN* genes also occur in young plants we examined the transcript levels of *OsPIN1* and *OsPIN3* in culm tissues of seed-germinated *RFL* partial knockdown *Ubi:RFL*(*as*)*-1* plants at different developmental stages. Comparative expression analysis was done using plants 15 and 30 days post-germination and in partial knockdown plants that had undergone the transition to flowering. We detect lowered transcript levels for *OsPIN1* and *OsPIN3* expression at all three growth stages (Fig. 5B), confirming the regulatory effect of *RFL* on their expression from early stages of AM outgrowth. To assess whether altered transcript levels for these auxin transporters influence hormone transport, we studied the effects of NPA, an inhibitor of auxin efflux pumps in root growth (Reed *et al.*, 1998). The effects on root growth in wild-type and *RFL* partial knockdown *Ubi:RFL*(*as*)*-1* plants were assessed. We observed that even without NPA treatment, seminal root length in *RFL* partial knockdown seedlings was significantly reduced as compared to wild-type seedlings. Wild-type seedlings subjected to NPA treatment show a statistically significant reduction in seminal root length, even at the lowest concentration of NPA used (Fig. 5C). On exposure to increasing concentrations of NPA, the roots of partial knockdown plants have a subtle reduction in root length that is statistically insignificant as compared to untreated partial knockdown plants (Fig. 5D). These data suggest compromised auxin transport in *RFL* partial knockdown plants, hence we examined the activity of the synthetic auxin-responsive reporter *IR4 DR5:GFP* in wild-type and *RFL* knockdown plants. This reporter is often used as a read-out for the spatially regulated buildup of auxin maxima that follows auxin transport by PIN proteins (Benkova *et al.*, 2003; Gallavotti *et al.*, 2008). In young regenerated wild-type plantlets with the *IR4 DR5:GFP* reporter cassette, which are expected to have a robust polar auxin transport system, we observed high levels of GFP florescence at the compact basal nodes, which contain the SAM and AMs (Supplementary Figure S6D). In contrast, *dsRNAiRFL* plantlets with a single T-DNA knockdown cassette and the *IR4 DR5:GFP* reporter have diffuse GFP florescence throughout the plantlet (Supplementary Figure S6 E), indicating poor basipetal auxin flow. Also, optical sections of shoot meristems in *IR4 DR5:GFP* transgenics show spatially restricted auxin maxima (Fig. 5F). On the other hand, a diffuse pattern of GFP activity is detected in apices of *RFL* knockdown plants (Fig. 5G). Further, we note that *dsRNAiRFL* culm tissues have a slight increase in *OsIAA7* and *OsIAA20* transcript levels (Fig. 5E). As the expression of these rice genes is auxin responsive (Jain *et al.*, 2006; Itoh *et al.*, 2008), our observations could reflect a deranged auxin buildup in *RFL* knockdown tissues.

We exploited the close phylogenetic relationship of AtPIN2, OsPIN2, and OsPIN3 (Viaene *et al.*, 2013) to detect OsPIN2/OsPIN3 proteins (Cho *et al.*, 2013) using cross-reacting antibodies (Supplementary Figure S6F; Supplementary Methods). Immunohistochemistry of wild-type culm tissues allowed the detection of signals in the vegetative SAM, and vascular strands in the leaf sheath and axillary buds (Supplementary Figure S6G–J). A subtle reduction is seen in the immunoreactivity of *amiR-RFL* shoot apices (Supplementary Figure S6I–K) and vascular tissues (Supplementary Figure S6G–J). These observations are supported by the reduced efficiency of ^3^H-IAA basipetal transport in stem and root tissues of *amiR-RFL* plants (Supplementary Table S2; Supplementary Methods). Together, these data suggest that the reduced expression of *OsPIN1* and *OsPIN3*, and the subtle defects in vascular tissues, contribute to weakened auxin transport in *RFL* partial knockdown plants.

### RFL modulates the expression of LAX1, CUC1, and OsPIN3

**Fig. 6.**
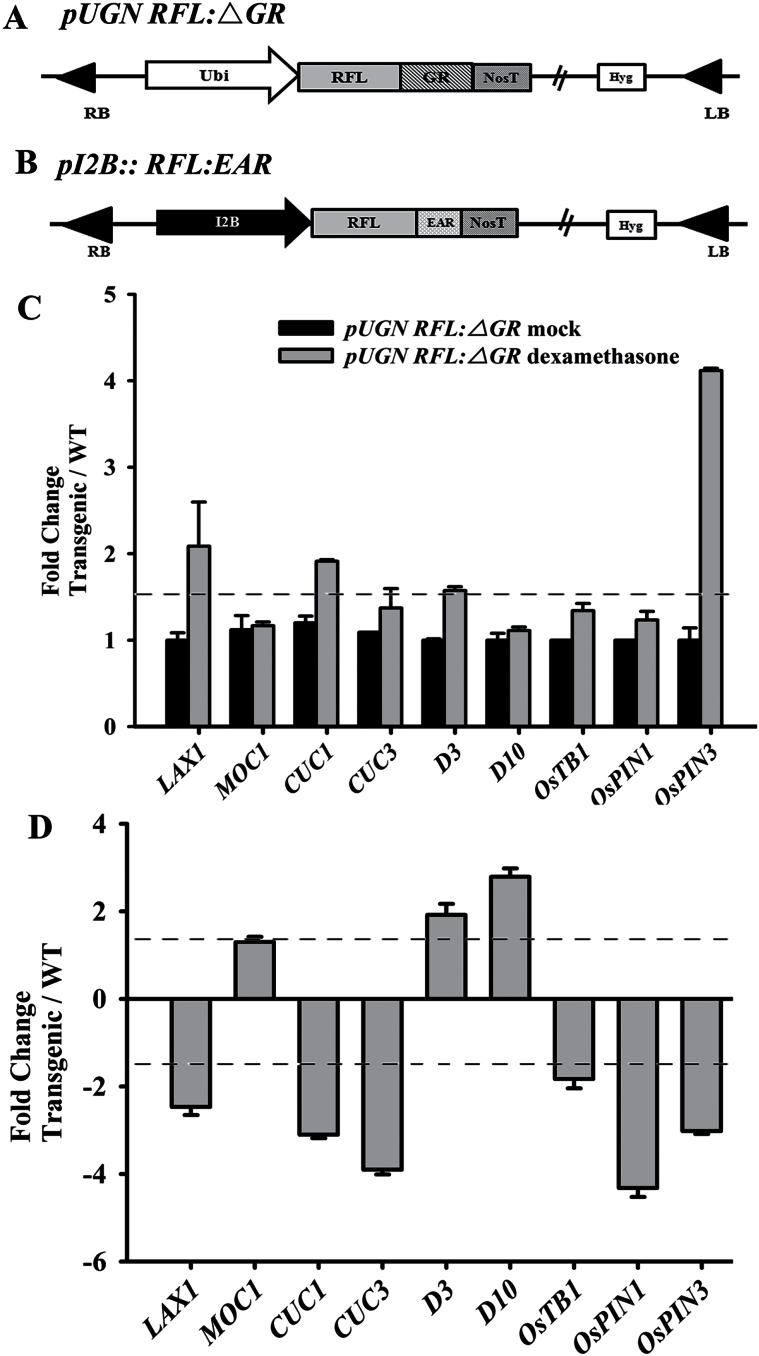
(A) Schematic representation of the T-DNA segment in the *pUGNRFL:GR* construct used for inducible ectopic expression of *RFL*. (B) Schematic representation of the T-DNA segment in the *pI2B::RFL:EAR* construct used for expression of a dominant repressive RFL form driven by minimal promoter and enhancer elements from the *RFL* locus. (C) RT-qPCR analyses of transcripts from candidate genes in the culm tissues of *pUGNRFL:GR* plants treated with dexamethasone or after mock treatment. Data is compared to that from culm tissues of similarly treated wild-type (WT) plants. Black bars indicate the fold change in transcript levels after mock treatment; grey bars indicate the fold change in transcript levels after dexamethasone treatment. (D) RT-qPCR analyses of candidate genes in culm tissues of *I2B::RFL:EAR* plants as compared to wild-type tissues, both harvested from plants after transition to flowering. The expression levels in C and D are represented as the mean of relative fold changes seen for three replicates from two independent biological samples. Error bars represent the standard error of the mean (*n* = 6). A fold change of 1.5 was taken to categorize genes regulated by *RFL* and is indicated by the dotted line in the graph.

To ascertain the ability of *RFL* to regulate the expression of *LAX1* and other AM development genes that were affected on knockdown of *RFL*, we created transgenics that express modified versions of the RFL protein. A binary construct, *pUGNRFL:GR*, where the T-DNA segment harbours an expression cassette for production of RFL protein with a C-terminal translational fusion to the GR domain, was made (Fig. 6A; Supplementary Methods). The fusion protein is chemically inducible in these transgenic plants. Under mock conditions, the *RFL:GR* protein would be cytoplasmically sequestered, while on dexamethasone treatment nuclear translocation and the downstream effects due to overexpression of RFL would occur. We raised 15 plants and used the T1 seeds from *pUGNRFL:GR-2* for further experimental analysis. Five plants were grouped to form two biological replicates and this was done for each of the 9h treatment regimes, i.e. with ethanol as the mock or with dexamethasone as the inducer. Wild-type plants were similarly mock or dexamethasone treated. RNA prepared from culm tissues of these groups of plants (wild type and *pUGNRFL:GR -2*) were used to determine expression levels of candidate genes involved in AM development. We noted about a 2-fold increase in the transcript levels for *LAX1* (Fig. 6C) and *CUC1* genes on the ectopic induction of RFL:GR. On the other hand, induced overexpression of *RFL:GR* had no effect on transcript levels for *MOC1* and *CUC3*. Importantly, *OsPIN3* expression was elevated up to 4-fold in culm tissues of *pUGNRFL:GR-2* plants that were dexamethasone treated (Fig. 6C), while no changes were noted in *OsPIN1* expression levels. The modulation in expression status of genes in the strigolactone signalling pathway was also examined, and here we studied effects on *D3*, *D10*, and *OsTB1* genes. We detected no significant change in their transcript levels on induction of *RFL*.

In a complementary approach, we undertook studies where the consequences of expressing a repressive form of *RFL* on gene expression in culm tissues could be examined. For these experiments we created a set of transgenic plants where the *cis* regulatory elements from the *RFL* locus (Prasad *et al.*, 2003) would drive the expression of RFL protein C-terminally tagged with the EAR transcription repressor motif (Fig. 6B; Hiratsu *et al.*, 2003; Supplementary Methods). Twelve transgenic plants, confirmed for the *I2B:RFL-EAR* T-DNA, were taken for comparative expression analysis with control wild-type regenerated plants. We observed that the transcript levels for *LAX1*, *CUC1*, *CUC3*, *OsTB1*, *OsPIN1*, and *OsPIN3* were reduced in the culm tissues of these plants that express the dominant repressive form of *RFL* (Fig. 6D). Together, these data show that *RFL* may directly modulate the transcript levels of *LAX1*, *CUC1* and *OsPIN3* in the culm, whereas the effects on strigolactone pathway genes and *OsPIN1* are probably indirect effects.

## Discussion

In this study, we examined the influence of rice *RFL* on factors that control specification and outgrowth of vegetative AMs. Our findings indicate that defects in AM development that occur on RFL knockdown arise from its regulatory effects first during specification of these meristems and later during their outgrowth as a tiller. Our data place meristem specification transcription factors *LAX1* and *CUC* downstream of *RFL*. Additionally, the positive regulatory effect of *RFL* on auxin transporters in the culm could affect outgrowth of AMs.

### RFL regulates genes important for AM specification

The *LAX1* gene is required for development of primary branches, secondary branches, and lateral spikelets of the rice panicle (Komatsu *et al.*, 2001). All these branch meristems are formed at the axils of bracts (Itoh *et al.*, 2005). While nearly all mutant alleles of *LAX1* show panicle-branching defects (Komatsu *et al.*, 2001), they have variable effects on tillering. The strong *lax1-2* null mutant, with reduced tillering, showed *LAX1* functions are relevant to all AMs (Oikawa and Kyozuka, 2009). In young vegetative tissues *RFL* transcripts are detected in leaf axils and in young AMs (Rao *et al.*, 2008). This expression domain encompasses cells that express *LAX1* mRNA and cells with the LAX1 protein (Oikawa and Kyozuka, 2009). We found a reduction of *RFL* activity caused persistent reduction in AM development even after plants had undergone the transition to flowering. Therefore, we examined the relationship between *RFL* and *LAX1* in the culm of plants at different developmental stages. We show that in the culm, *LAX1* transcript levels are co-related with those of *RFL*. Further, our data from gene expression effects in transgenics with repressive or inducible *RFL* show modulation in *LAX1* transcript levels. Recent studies on the rice *lax2* mutant indicate that multiple pathways contribute to AM development, as the *lax1 lax2* double mutants have synergistic tillering defects (Tabuchi *et al.*, 2011). While LAX2, a novel nuclear protein, does not affect expression of LAX1, it is an interacting partner proposed to contribute to the AM functions of LAX1. Our findings imply a probable direct activating role for *RFL* in AM development that acts, in part, through attaining appropriate *LAX1* expression levels. These findings are analogous to the role ascribed to a maize AT-hook protein, BAF1 that, along with unknown factors, was required for threshold levels of *BA1* (maize *LAX1* orthologue) expression (Galavotti *et al*., 2011). *BAF1* thereby contributed to AM initiation in maize either upstream of, or parallel to, *BA1*. *Arabidopsis LFY* has a predominant role in conferring floral meristem (FM) identity (Weigel *et al.*, 1992; Wagner, 2009; Moyroud *et al.*, 2010) with its functions in AMs being unclear until recently. The latter functions were uncovered with the new *LFY*_*HARA*_ allele with only partial defects in FM identity (Chahtane *et al.*, 2013). This mutant allele showed that LFY can promote growth of vegetative AMs through its direct target *REGULATOR OF AXILLARY MERISTEMS1* (*RAX1*), encoding an R2R3 MYB domain factor (Chahtane *et al.*, 2013). These functions for *Arabidopsis LFY* and *RAX1* in AM development are parallel to, and redundant with, the pathway regulated by *LATERAL SUPPRESSOR* (*LAS*) and *REGULATOR OF AXILLARY MERISTEM FORMATION1* (*ROX1*). The latter gene activities are largely functional only in AMs and not FMs (Greb *et al.*, 2003; Yang *et al.*, 2012). Interestingly, *ROX1* is orthologous to rice *LAX1* and we find that *LAX1* expression levels in flowering panicles and vegetative AMs is dependent on the expression status of *RFL* (Rao *et al.*, 2008; also this study). Our data also show elevated transcript levels for *MOC1*, the homologue of *Arabidopsis LAS*, in plants downregulated for *RFL*. However, we found that induction of RFL:GR and expression of the repressive RFL–EAR protein did not significantly alter *MOC1* transcript levels. Therefore, we postulate that in *RFL* knockdown plants the changes in *MOC1* transcript levels arise due to indirect consequences. Further, as *MOC1* is subjected to post-translational and post-transcriptional control (Lin *et al.*, 2012; Xu *et al.*, 2012), understanding any relationship between *RFL* and *MOC1* requires further studies. Thus, we speculate that the multiple pathways that control *Arabidopsis* AM development, i.e. LFY-dependent and LFY-independent mechanisms, are closely linked in rice.

In *Arabidopsis*, *CUC2* and *CUC3* genes, in addition to their role in shoot meristem formation and organ separation, play a role in AM development possibly by defining a boundary for the emerging AM. These functions for the *Arabidopsis CUC* genes are routed through their effects on *LAS* and by mechanisms that are independent of *LAS* (Hibara *et al.*, 2006; Raman *et al.*, 2008). A relationship between *Arabidopsis LFY* and *CUC* genes in AMs has not yet been defined, but the direct regulatory effects of *LFY* on *CUC2* in FMs is known (Winter *et al.*, 2011). Our data show modulation in *RFL* activity leads to corresponding expression changes in *CUC1*, *CUC2*, and *CUC3* gene expression in the culm. Further, we note overlapping expression domains for rice *RFL*, *OsNAM/CUC2*, and *OsCUC3* in leaf axils (Hibara and Nagato, 2006; Rao *et al.*, 2008). Thus, during rice AM development, the meristem functions of *RFL* and *CUC* genes are related.

### RFL regulates AM outgrowth by positively influencing auxin transport

The outgrowth of axillary buds is also influenced by hormonal signals and external cues such as planting density, nutrient availability, and light (Shimizu-Sato and Mori 2001; Beveridge *et al.*, 2003; Leyser, 2003; Brewer *et al.*, 2013). Studies of *Arabidopsis max*, pea *rms*, and rice *dwarf* strigolactone mutants provide two mutually non-exclusive models that explain the general relationships between auxin, strigolactone, and bud outgrowth, but details of temporal and spatial dynamics of these links are unclear. One model postulates that strigolactones inhibit bud outgrowth by altering gene expression with possible direct effects on AMs (Brewer *et al.*, 2009; Brewer *et al.*, 2013). Computational modelling of long-distance auxin transport and decapitation experiments with pea support this model, and suggest that strigolactone may affect bud meristem cell division downstream of *BRC1*, a TCP-domain transcription factor (Brewer *et al.*, 2009; Dun *et al.*, 2012; Renton *et al.*, 2012). Other recent reports using *Arabidopsis* show that strigolactones inhibit bud outgrowth by depleting plasma membrane-localized PIN transporters (Crawford *et al.*, 2010; Shinohara *et al.*, 2013). These findings form the mechanistic basis for the second model, which proposes that strigolactone inhibits bud outgrowth depending on the overall auxin transport status of the plant, with bud outgrowth requiring active export of auxin.

Here, we have shown that transgenic plants compromised for *RFL* function have reduced levels of *OsPIN1* and *OsPIN3* transcripts and diffuse auxin-sensitive reporter activity in the SAM (Fig. 5). Supporting this are immunolocalization data that hint at a mild reduction in OsPIN2/OsPIN3 cross-reactivity in *RFL* knockdown plants, further supported by impaired auxin transport in the plants (Fig. 5; Supplementary Figure S6; Supplementary Table S2). Our findings are consistent with a relationship between *RFL* and auxin transport in vegetative culms during bud outgrowth, with probable direct effects on *OsPIN3.* We cannot exclude the effects of *RFL* on transporters such as those of the ABCB family (Multani *et al.*, 2003; Knöller *et al.*, 2010). Other likely players downstream of *RFL* could be factors like AXR1 (Stirnberg *et al*, 1999); their contributions remain to be tested. The indirect regulatory effects of *RFL* on bud-specific transcription factor genes such as *OsTB1* could also contribute to outgrowth.

Overall, our studies indicate an important role for *RFL* in vegetative AM specification and perhaps on the outgrowth of axillary buds.

## Supplementary material

Supplementary Table S1. Primers used in this study.

Supplementary Table S2. PATS assay in wild-type and *amiR-RFL* plants.

Supplementary Figure S1. Expression of hairpin loop RNA in *dsRNAiRFL* plants detected by RT-PCR analysis of transgenic leaf tissues.

Supplementary Figure S2. Transcript levels for *D3* and *RFL* in various rice tissues.

Supplementary Figure S3. Copy number determination for T-DNAs in *Ubi amiRD3*, *dsRNAiRFL* and *amiR D3-dsRNAiRFL* lines.

Supplementary Figure S4. Plant height phenotype for *dsRNAiRFL*, *Ubi amiRD3*, *amiR D3-dsRNAiRFL*, and wild-type plants.

Supplementary Figure S5. Effect of GR24 on transcript levels of *OsPIN1* and *OsPIN3* in wild-type plants of three developmental growth stages.

Supplementary Figure S6. Live imaging of GFP reporter activity driven by an auxin-responsive promoter in young *dsRNAiRFL* and wild-type plantlets; and immunohistochemistry for OsPIN2/OsPIN3 in histological sections of wild-type and *amiR RFL* culm tissues.

Supplementary Methods. These methods are for the design of various constructs; live imaging; genomic DNA isolation and determination of T-DNA copy number; immunoblotting and immunohistochemistry; GR24 treatment; dexamethasone-based induction; and PATS assay.

Supplementary Data

## References

[CIT0001] Aida M Ishida T Fukaki H Fujisawa H Tasaka M 1997 Genes involved in organ separation in *Arabidopsis*: an analysis of the cup-shaped cotyledon mutant. The Plant Cell 9, 841–857. 9212461 10.1105/tpc.9.6.841 PMC156962

[CIT0002] Arite T Iwata H Ohshima K 2007 *DWARF10*, an *RMS1*/*MAX4*/*DAD1* ortholog, controls lateral bud outgrowth in rice. The Plant Journal 51, 1019–1029. 17655651 10.1111/j.1365-313X.2007.03210.x

[CIT0003] Arite T Umehara M Ishikawa S Hanada A Maekawa M Yamaguchi S Kyozuka J 2009 *d14*, a strigolactone-insensitive mutant of rice, shows an accelerated outgrowth of tillers. Plant Cell Physiology 50, 1416–1424. 19542179 10.1093/pcp/pcp091

[CIT0004] Benkova E Michniewicz M Sauer M Teichmann T Seifertova D Jürgens G Friml J 2003 Local, efflux-dependent auxin gradients as a common module for plant organ formation. Cell 115, 591–602. 14651850 10.1016/s0092-8674(03)00924-3

[CIT0005] Beveridge CA Weller JL Singer SR Hofer JM 2003 Axillary meristem development. Budding relationships between networks controlling flowering, branching, and photoperiod responsiveness. Plant Physiology 131, 927–934. 12644645 10.1104/pp.102.017525 PMC1540292

[CIT0006] Booker J Sieberer T Wright W 2005 *MAX1* encodes a cytochrome P450 family member that acts downstream of *MAX3/4* to produce a carotenoid-derived branch-inhibiting hormone. Developmental Cell 8, 443–449. 15737939 10.1016/j.devcel.2005.01.009

[CIT0007] Brewer B Koltai H Beveridge CA 2013 Diverse roles of strigolactones in plant development. Molecular Plant 6, 18–28. 23155045 10.1093/mp/sss130

[CIT0008] Brewer PB Dun EA Ferguson BJ Rameau C Beveridge CA 2009 Strigolactone acts downstream of auxin to regulate bud outgrowth in pea and *Arabidopsis* . Plant Physiology 150, 482–493. 19321710 10.1104/pp.108.134783 PMC2675716

[CIT0009] Chahtane H Vachon G Le Masson M 2013 A variant of *LEAFY* reveals its capacity to stimulate meristem development by inducing *RAX1* . The Plant Journal 74, 678–689. 23445516 10.1111/tpj.12156

[CIT0010] Cho SH Yoo SC Zhang H Pandeya D Koh HJ Hwang JY Kim GT Paek NC 2013 The rice narrow leaf2 and narrow leaf3 loci encode WUSCHEL-related homeobox 3A (OsWOX3A) and function in leaf, spikelet, tiller and lateral root development. New Phytologist 198, 1074–1084. 10.1111/nph.12231 23551229

[CIT0011] Crawford S Shinohara N Sieberer T Williamson L George G Hepworth J Muller D Domagalska MA Leyser O 2010 Strigolactones enhance competition between shoot branches by dampening auxin transport. Development 137, 2905–2913. 20667910 10.1242/dev.051987

[CIT0012] Dun A Germain A Rameau C Beveridge CA 2012 Antagonistic action of strigolactone and cytokinin in bud outgrowth control. Plant Physiology 158, 487–498. 22042819 10.1104/pp.111.186783 PMC3252097

[CIT0013] Gallavotti A Malcomber S Gaines C Stanfield S Whipple C Kellogg E Schmidt RJ 2011 BARREN STALK FASTIGIATE1 is an AT-hook protein required for the formation of maize ears. The Plant Cell 23, 1756–1771. 21540434 10.1105/tpc.111.084590 PMC3123940

[CIT0014] Gallavotti A Yang Y Schmidt RJ Jackson D 2008 The relationship between auxin transport and maize branching. Plant Physiology 147, 1913–1923. 18550681 10.1104/pp.108.121541 PMC2492655

[CIT0015] Gomez-Roldan V Fermas S Brewer PB 2008 Strigolactone inhibition of shoot branching. Nature 455, 189–194. 18690209 10.1038/nature07271

[CIT0016] Greb T Clarenz O Schäfer E Muller D Herrero R Schmitz G Theres K 2003 Molecular analysis of the *LATERAL SUPPRESSOR* gene in *Arabidopsis* reveals a conserved control mechanism for axillary meristem formation. Genes and Development 17, 1175––1187. 12730136 10.1101/gad.260703 PMC196050

[CIT0017] Hibara K Karim MR Takada S Taoka K Furutani M Aida M Tasaka M 2006 *Arabidopsis CUP-SHAPED COTYLEDON3* regulates postembryonic shoot meristem and organ boundary formation. The Plant Cell 18, 2946–2957. 17122068 10.1105/tpc.106.045716 PMC1693926

[CIT0018] Hibara K Nagato Y 2006 *OsNAM* and *OsCUC3* are expressed specifically in organ boundaries. Rice Genetics Newsletter 23, 96–97.

[CIT0019] Hiratsu K Matsui K Koyama T Ohme-Tahasi M 2003 Dominant repression of target genes by chimeric repressors that include the EAR motif, a repression domain, in *Arabidopsis* . The Plant Journal 34, 733–739. 12787253 10.1046/j.1365-313x.2003.01759.x

[CIT0020] Ikeda-Kawakatsu K Maekawa M Izawa T Itoh J Nagato Y 2012 *ABERRANT PANICLE ORGANIZATION 2/RFL*, the rice ortholog of *Arabidopsis LEAFY*, suppresses the transition from inflorescence meristem to floral meristem through interaction with *APO1* . The Plant Journal 69, 168–180. 21910771 10.1111/j.1365-313X.2011.04781.x

[CIT0021] Ishikawa S Maekawa M Arite T Onishi K Takamure I Kyozuka J 2005 Suppression of tiller bud activity in tillering dwarf mutants of rice. Plant Cell Physiology 46, 79–86. 15659436 10.1093/pcp/pci022

[CIT0022] Itoh JI Hibara KI Sato Y Nagato Y 2008 Developmental role and auxin responsiveness of class III homeodomain leucine zipper gene family members in rice. Plant Physiology 147, 1960–1975. 18567825 10.1104/pp.108.118679 PMC2492597

[CIT0023] Itoh J Nonomura K Ikeda K Yamaki S Inukai Y Yamagishi H Kitano H Nagato Y 2005 Rice plant development: from zygote to spikelet. Plant Cell Physiology 46, 23–47. 15659435 10.1093/pcp/pci501

[CIT0024] Jain M Kaur N Garg R Thakur JK Tyagi AK Khurana JP 2006 Structure and expression analysis of early auxin-responsive *Aux/IAA* gene family in rice (Oryza sativa). Functional and Integrative Genomics 6, 47–59. 16200395 10.1007/s10142-005-0005-0

[CIT0025] Jiang L Liu X Xiong G 2013 *DWARF 53* acts as a repressor of strigolactone signalling in rice. Nature 504, 401–405. 24336200 10.1038/nature12870 PMC5802366

[CIT0026] Knöller AS Blakeslee JJ Richards EL Peer WA Murphy AS 2010 Brachytic2/ZmABCB1 functions in IAA export from intercalary meristems. Journal of Experimental Botany 61, 3689–3696. 20581123 10.1093/jxb/erq180 PMC2921204

[CIT0027] Komatsu K Maekawa M Ujiie S Satake Y Furutani I Okamoto H Shimamoto K Kyozuka J 2003 *LAX* and *SPA*: Major regulators of shoot branching in rice. Proceedings of National Academy of Sciences, USA 100, 11765–11770. 10.1073/pnas.1932414100 PMC208832 13130077

[CIT0028] Komatsu M Maekawa M Shimamoto K Kyozuka J 2001 The *LAX1* and *FRIZZYPANICLE 2* genes determine the inflorescence architecture of rice by controlling rachis-branch and spikelet development. Developmental Biology 231, 364–373. 11237465 10.1006/dbio.2000.9988

[CIT0029] Kyozuka J Konishi S Nemoto K Izawa T Shimamoto K 1998 Down regulation of *RFL* the *FLO*/*LFY* homology of rice accompanied with panicle and branch initiation. Proceedings of National Academy of Sciences, USA 95, 1979–1982. 10.1073/pnas.95.5.1979 PMC33826 9482818

[CIT0030] Leyser O 2003 Regulation of shoot branching by auxin. Trends in Plant Science 8, 541–545. 14607099 10.1016/j.tplants.2003.09.008

[CIT0031] Liang W Shang F Lin Q Lou C Zhang J 2014 Tillering and panicle branching genes in rice. Gene 537, 1–5. 24345551 10.1016/j.gene.2013.11.058

[CIT0032] Lin H Wang R Qian Q 2009 *DWARF27*, an iron-containing protein required for the biosynthesis of strigolactones, regulates rice tiller bud outgrowth. The Plant Cell 21, 1512–1525. 19470589 10.1105/tpc.109.065987 PMC2700539

[CIT0033] Lin Q Wang D Dong H 2012 Rice APC/C(TE) controls tillering by mediating the degradation of *MONOCULM 1* . Nature Communications 3, 752. 10.1038/ncomms1716 PMC3316886 22434195

[CIT0034] Li X Qian Q Fu Z 2003 Control of tillering in rice. Nature 422, 618–621. 12687001 10.1038/nature01518

[CIT0035] Minakuchi K Kameoka H Yasuno N 2010 *FINE CULM1* (*FC1*) works downstream of strigolactones to inhibit the outgrowth of axillary buds in rice. Plant Cell Physiology 51, 1127–1135. 20547591 10.1093/pcp/pcq083 PMC2900823

[CIT0036] Moyroud E Kusters E Monniaux M Koes R Parcy F 2010 *LEAFY* blossoms. Trends in Plant Science 6, 346–352. 20413341 10.1016/j.tplants.2010.03.007

[CIT0037] Multani DS Briggs SP Chamberlin MA Blakeslee JJ Murphy AS Johal GS 2003 Loss of an MDR Transporter in Compact Stalks of Maize *br2* and Sorghum *dw3* mutants. Science 302, 81–83. 14526073 10.1126/science.1086072

[CIT0038] Oikawa T Kyozuka J 2009 Two-step regulation of LAX PANICLE1 protein accumulation in axillary meristem formation in rice. The Plant Cell 21, 1095–1108. 19346465 10.1105/tpc.108.065425 PMC2685638

[CIT0039] Pautler M Tanaka W Hirano HY Jackson D 2013 Grass meristem I. Shoot apical meristem maintenance, axillary meristem determinacy and the floral transition. Plant Cell Physiology 54, 302–312. 23411664 10.1093/pcp/pct025

[CIT0040] Prasad K Kushalappa K Vijayraghavan U 2003 Mechanism underlying regulated expression of *RFL*, a conserved transcription factor, in the developing rice inflorescence. Mechanisms of Development 120, 491–502. 12676326 10.1016/s0925-4773(02)00457-4

[CIT0041] Prasad K Sriram P Kumar CS Kushalappa K Vijayraghavan U 2001 Ectopic expression of rice *OsMADS1* reveals a role in specifying the lemma and palea, grass floral organs analogous to sepals. Development Genes and Evolution 211, 281–290. 11466523 10.1007/s004270100153

[CIT0042] Raman S Greb T Peaucelle A Blein T Laufs P Theres K 2008 Interplay of miR164, *CUP-SHAPED COTYLEDON* genes and *LATERAL SUPPRESSOR* controls axillary meristem formation in *Arabidopsis thaliana* . The Plant Journal 55, 65–76. 18346190 10.1111/j.1365-313X.2008.03483.x

[CIT0043] Rao NN Prasad K Kumar PR Vijayraghavan U 2008 Distinct regulatory role for *RFL*, the rice *LFY* homolog, in determining flowering time and plant architecture. Proceedings of the National Academy of Sciences, USA 105, 3646–3651. 10.1073/pnas.0709059105 PMC2265177 18305171

[CIT0044] Reed RC Brady SR Muday GK 1998 Inhibition of auxin movement from the shoot into the root inhibits lateral root development in *Arabidopsis* . Plant Physiology 118, 1369–1378. 9847111 10.1104/pp.118.4.1369 PMC34753

[CIT0045] Reinhardt D Mandel T Kuhlemeier C 2000 Auxin regulates the initiation and radial position of plant lateral organs. The Plant Cell 12, 507–518. 10760240 10.1105/tpc.12.4.507 PMC139849

[CIT0046] Renton M Hanan J Ferguson J Beveridge CA 2012 Models of long-distance transport: how is carrier-dependent auxin transport regulated in the stem? New Phytologist 194, 704–715. 22443265 10.1111/j.1469-8137.2012.04093.x

[CIT0047] Sentoku N Sato Y Kurata N Ito Y Kitano H Matsuoka 1999 Regional expression of the rice *KN1*-type homeobox gene family during embryo, shoot, and flower development. The Plant Cell 11, 1651–1663. 10488233 10.1105/tpc.11.9.1651 PMC144314

[CIT0048] Shen H Luong P Huq E 2007 The F-box protein MAX2 functions as a positive regulator of photomorphogenesis in *Arabidopsis* . Plant Physiology 145, 1471–1483. 17951458 10.1104/pp.107.107227 PMC2151697

[CIT0049] Shimizu-Sato S Mori H 2001 Control of outgrowth and dormancy in axillary buds. Plant Physiology 127, 1405–1413. 11743082 PMC1540171

[CIT0050] Shinohara N Taylor C Leyser O 2013 Strigolactone can promote or inhibit shoot branching by triggering rapid depletion of the auxin efflux protein PIN1 from the plasma membrane. PLoS Biology 11, e1001474. 23382651 10.1371/journal.pbio.1001474 PMC3558495

[CIT0051] Stirnberg P Chatfield SP Leyser HMO 1999 *AXR1* Acts after Lateral Bud Formation to Inhibit Lateral Bud Growth in *Arabidopsis.* Plant Physiology 121, 839–847. 10557232 10.1104/pp.121.3.839 PMC59446

[CIT0052] Stirnberg P van de Sande K Leyser O 2002 *MAX1* and *MAX2* control shoot lateral branching in *Arabidopsis* . Development 129, 1131–1141. 11874909 10.1242/dev.129.5.1131

[CIT0053] Tabuchi H Zhang Y Hattori S 2011 *LAX PANICLE2* of rice encodes a novel nuclear protein and regulates the formation of axillary meristems. The Plant Cell 23, 3276– –3287. 21963665 10.1105/tpc.111.088765 PMC3203427

[CIT0054] Umehara M Hanada A Yoshida S 2008 Inhibition of shoot branching by new terpenoid plant hormones. Nature 455, 195–200. 18690207 10.1038/nature07272

[CIT0055] Viaene Delwiche CF Rensing SA Friml J 2013 Origin and evolution of PIN auxin transporters in the green lineage. Trends in Plant Science 18, 5–10. 22981345 10.1016/j.tplants.2012.08.009

[CIT0056] Vroemen CW Mordhorst AP Albrecht C Kwaaitaal MA de Vries SC 2003 The *CUP-SHAPED COTYLEDON3* gene is required for boundary and shoot meristem formation in *Arabidopsis* . The Plant Cell 15, 1563–1577. 12837947 10.1105/tpc.012203 PMC165401

[CIT0057] Wagner D 2009 Flower morphogenesis: timing is key. Development Cell 16, 621–622. 10.1016/j.devcel.2009.05.005 19460335

[CIT0058] Weigel D Alvarez J Smyth DR Yanofsky MF Meyerowitz EM 1992 *LEAFY* controls floral meristem identity in *Arabidopsis* . Cell 69, 843–859. 1350515 10.1016/0092-8674(92)90295-n

[CIT0059] Winter CM Austin RS Blanvillain-Baufume S 2011 *LEAFY* target genes reveal floral regulatory logic, *cis* motifs, and a link to biotic stimulus response. Developmental Cell 20, 430–443. 21497757 10.1016/j.devcel.2011.03.019

[CIT0060] Wisniewska J Xu J Seifertova D Brewer PB Ruzicka K Blilou I Rouquie D Benkova E Scheres B Friml J 2006 Polar PIN localization directs auxin flow in plants. Science 312, 883. 16601151 10.1126/science.1121356

[CIT0061] Xu C Wang Y Yu Y Duan J Liao Z Xiong G Meng X Liu G Qian Q Li J 2012 Degradation of MONOCULM 1 by APC/C(TAD1) regulates rice tillering. Nature Communications 3, 750. 10.1038/ncomms1743 PMC3316885 22434193

[CIT0062] Xu M Zhu L Shou H Wu P 2005 A PIN1 family gene, *OsPIN1*, involved in auxin-dependent adventitious root emergence and tillering in rice. Plant Cell Physiology 46, 1674–1681. 16085936 10.1093/pcp/pci183

[CIT0063] Yadav SR Prasad K Vijayraghavan U 2007 Divergent regulatory *OsMADS2* functions control size, shape and differentiation of the highly derived rice floret second-whorl organ. Genetics 176, 283–294. 17409064 10.1534/genetics.107.071746 PMC1893039

[CIT0064] Yang F Wang Q Schmitz G Muller D Theres K 2012 The bHLH protein ROX acts in concert with RAX1 and LAS to modulate axillary meristem formation in *Arabidopsis* . The Plant Journal 71, 61–70. 22372440 10.1111/j.1365-313X.2012.04970.x

[CIT0065] Zou J Zhang S Zhang W 2006 The rice *HIGH -TILLERING DWARF1* encoding an ortholog of Arabidopsis *MAX3* is required for negative regulation of the outgrowth of axillary buds. The Plant Journal 48, 687–698. 17092317 10.1111/j.1365-313X.2006.02916.x

