# Supplementary material for: Functions for rice RFL in vegetative axillary meristem specification and outgrowth
Source: J Exp Bot. 2015 Mar 18;66(9):2773–84. doi: 10.1093/jxb/erv092 (PMC4986878; doi:10.1093/jxb/erv092)
Supplement: Supplementary Data [file supp_66_9_2773__index.html]

Functions for rice RFL in vegetative axillary meristem specification and outgrowth — Functions for rice RFL in vegetative axillary meristem specification and outgrowth — Supplementary Data 

# Functions for rice RFL in vegetative axillary meristem specification and outgrowth

## Supplementary Data

Data files

**Files in this Data Supplement:**

- Supplementary Data - Supplementary Data
