# Supplementary material for: Functions for rice RFL in vegetative axillary meristem specification and outgrowth
Source: J Exp Bot. 2015 Mar 18;66(9):2773–84. doi: 10.1093/jxb/erv092 (PMC4986878; doi:10.1093/jxb/erv092)

Journal of Experimental Botany

Title: Functions for rice RFL in vegetative axillary meristem specification and outgrowth

Gauravi M. Deshpande, Kavitha Ramakrishna, Grace L Chongloi and Usha Vijayraghavan

SUPPLEMENTARY DATA

Supplemental Methods

Constructs for Artificial miRNA-based Knockdown of *RFL*

An artificial miRNA specific for endogenous *RFL* was designed targeting its 3'UTR using the web tool (http://wmd2.weigelworld.org/cgi-bin/mirnatools.plpage=2). The chosen *RFL* sequences aggcagttaggtcgttctaagc, lie 83 nts downstream of the stop codon. Four primers named amiR RFL I, II, III, and IV were used for PCRs with two pNW55 vector specific primers G-4368 and G4369, in the combination G-4368+II, I+IV and III+ G-4369 as per Schwab *et al.* (2006). This yielded 256, 87 and 259 bps amplicons which were used for the overlap PCR to obtain a 554 bps amplicon fragment in which the original osa-MIR528 sequences in pNW55 could be replaced by *RFL* amiRNA. This 554 bps fragment was cloned at EcoRV site of pBSKS from which a 255 bp BamHI– KpnI fragment encoding the precursor miRNA was then cloned into plant binary vector pUN to create *Ubi amiR RFL.*

Constructs for Artificial miRNA-based Knockdown of *D3* in wild type and *RFL* knockdown genetic backgrounds

An artificial miRNA specific for *D3* was designed against its 3'UTR using the web tool (http://wmd2.weigelworld.org/cgi-bin/mirnatools.plpage=2). The nucleotide sequences chosen for knockdown of *D3* - TAGGCTTATCGCCTTGTATAA, corresponded to 3415-3435 nts of its 3'UTR, 1.16 Kb downstream of the stop codon. Four primers named amiR D3 I, II, III, and IV (Supplementary Table S1 for primer sequence) were used for PCRs with two pNW55 vector specific primers G-4368 and G4369, in the combination G-4368+II, I+IV and III+ G-4369 to yield 256, 87 and 259 bps amplicons. The resulting fragments were used for the overlap PCR to

yield 554 bps fragment in which the original osa-MIR528 present in pNW55 are replaced by D3 amiRNA. This fragment was cloned at EcoRV site of pBSKS (*pBSKS D3amiRNA* #3). Subsequently the 255 bps BamHI- KpnI fragment encoding precursor mRNA was cloned into pUN vector cut with BamHI- KpnI, creating *pUbi amiR D3 nosT* construct.

The 255 bps D3 pre-artificial miRNA in pBSKS (pBSKS D3amiRNA #3) was also cloned under CaMV promoter. For this the 255 bps D3 pre-amiRNA was released as BamHI- KpnI and cloned into into pUC19CaMV NOS. The 1.2 Kb expression cassette containing CaMV promoter, amiR D3 and Nos terminator was then released as HindIII- EcoRI and end-filled using Klenow DNA polymerase and cloned into PmeI site of the binary vector *dsRNAiRFL*. The later construct had an expression cassette for hairpin loop RNAs, against *RFL* expressed from ubiquitin promoter. This final binary vector thus created was *dsRNAiRFL-CaMVamiRD3nosT*.

Constructs for expression of inducible and repressive forms of *RFL*

An ectopically inducible form of RFL was created by C terminal translational fusion with glucocorticoid receptor (ΔGR) domain. A 1.2 Kb fragment containing RFL coding sequences was excised with BglII from plasmid pRSET B RFL PvuII-BglII and was cloned at the BamHI site upstream of ΔGR domain in the vector pUGN. The later had the Ubiquitin promoter followed by a fragment encoding Glucocorticoid receptor domain and the Nos terminator. The final binary construct was named as *pUGN RFL: ΔGR*.

A dominant repressive form of *RFL* was created by the fusion of EAR domain C terminal to RFL. The plasmid pRSETB -RFLE1E2E3 #O with the full length RFL coding sequences was taken as the template for amplification using T7 FP and a RFLEAR RP primers (Supplementary Table S1 for primer details). The resulting PCR amplicon of 1.5 Kb was first cloned into the EcoRV site of pBSKS vector (pBSKS RFL:EAR#24). Then the 1.5 Kb SmaI- KpnI fragment containing *RFL* translationally fused to EAR was cloned into pUN vector at SmaI- KpnI sites. This created *pUbiRFL:EAR* construct. The ubiquitin promoter in *pUbi RFL:EAR* was replaced with a 1.9 Kb fragment of I2B *cis* sequence from *RFL* locus. The latter was amplified using I2B FP and I2B RP on I2B::GUS (Prasad *et al*., 2003). (Supplementary Table S1 for primer details). This created *pI2B::RFL:EAR*.

Constructs for imaging analysis of auxin pattern in wild type and knock-down of *RFL* genetic backgrounds

To generate promoter::reporter constructs to map the pattern of auxin, a synthetic auxin-responsive promoter IR4DR5 (a kind gift from Prof. Ben Scheres, Utrecht University, Netherlands) was transcriptionally fused to GFP as an EcoRI fragment. The 1.5Kb *IR4DR5::GFP NosT* was excised as a KpnI-XbaI fragment and cloned at corresponding sites of the plant transformation vector pCAMBIA1300 and named *IR4DR5:GFP*. Also, the same construct was cloned as a PmeI fragment into the plant binary vector *dsRNAiRFL* to finally create *dsRNAiRFL-IR4DR5:GFP*.

Live imaging

Transgenic plants containing *IR4DR:GFP* and *dsRNAiRFL-IR4DR5:GFP* young seedlings of (10 days post transfer to soilrite) were imaged using Olympus Stereomicroscope SZX16 equipped with SDFPLAPO1XPF air objective and a GFP Filter. Images were further processed in Adobe Photoshop.

Genomic DNA isolation and determination of T-DNA copy number of transgenics

Total genomic DNA was isolated from leaf tissues as described in Saghai Maroof *et al.* (1984). Genomic DNA qPCRs were carried out to determine the transgene copy number using 25 ng of genomic DNA and primers for the hygromycin cassette in the T-DNA segment of transgenic plants. qPCR using primers specific for actin, an endogenous single copy gene in the rice genome, served as a normalization control. Single copy T-DNA insertion rice transgenic line *pUbi::amiR D3-4*, double copy T-DNA insertion in line *pUbi::amiR D3-1* and three copies of T-DNA insertion in line *IR4DR5::GFP -5* (generated in the lab) that were independently verified by southern blot analysis, was used as a reference control in the qPCR based detection of single copy T-DNA insertions in other lines.

Immunoblotting and Immunohistochemistry

To examine the cross-reactivity of Anti-AtPIN2 (Agrisera, AS05069) to rice OsPIN3, a 1305 bp cDNA fragment was amplified from the cDNA clone - AK101504 , procured from RGRC (http://www.dna.affrc.go.jp/distribution/). A 1404bp cDNA fragment of *AtPIN2* was amplified on cDNA synthesized from 8 day old *Arabidopsis thaliana* (Col) seedlings. These amplified products were transcribed with T7 RNA polymerase and the mRNA taken for *in vitro* translation using Rabbit Reticulocyte Lysate System (Promega, Cat#L4960) as per manufacturer's instructions. The translated proteins were separated on an SDS-PAGE gel and transferred to PVDF membrane (Amersham). The membrane was blocked in Tris buffered saline with 3% BSA and then reacted with Anti-AtPIN2 (1:1000, Agrisera, AS05069) and further probed with1:5000 Donkey anti-chicken IgG HRP conjugated secondary antibody (703-035-155, Jackson Immuno Research Laboratories). Signals were developed using Super Signal West Pico Chemiluminescent Substrate (Millipore).

For Immunohistochemistry on rice tissues, 8µm sections were dewaxed in xylene, rehydrated and blocked in 5% non-fat dry milk in PBS then probed with 1:200 dilution of Anti- At PIN2 (Agrisera, AS05069). Preadsorbed secondary antibody - Goat anti-chicken IgY TriTC (103-025-155, Jackson Immuno Research Laboratories) was used at a dilution of 1:750. Washes were performed in 1xPBST and images were captured in Zeiss LSM confocal microscope at 10% laser power, Ex/Em of 540/570. Images were processed with Image J (http://imagej.nih.gov/ij/) and assembled in Adobe Photoshop. Mean fluorescence intensity was analyzed from 3 different areas. Anti-PIN2 and Goat Anti-chicken IgY TriTC were a kind a gift from Prof.M.K Matthew, NCBS, India. Donkey Anti-chicken IgG HRP conjugated antibody was a kind gift from Dr. Colin Jamora, InStem-Bangalore, India.

GR24 treatment

The treatment regime for strigolactone analog GR24 was per Umehara *et al*. (2008), with some modifications. 25 plants each for the age groups 15 day, 30 day old and plants that had

undergone transition (with ~0.2 cms panicles) were acclimatized in hydroponics medium for three days in a growth chamber held at 24 °C and in 16 h/8 h light/dark cycles. Plants were supplemented with 1 μM, 2 μM and 3 μM concentration of GR24 (Chiralix) for 24 h in the hydroponics medium and equal concentration of acetone was given as control treatment. For the long term treatment, the hydroponics medium was changed every 48 hrs and was continued till the bud outgrowth was seen. After 24h leaves were removed and vegetative culms of 15 day and 30 day plants were harvested for RNA isolation. For the plants after flowering transition, axillary meristem tissues in the culm were collected and taken for RNA isolation. RNA was isolated by Trizol method (TRI reagent, Sigma) as per manufacturer's instructions. 5 plants were grouped for each 24 h treatment so as to form two biological replicates with controls. For the longer term 20 days of GR24 treatment experiment was continued in the hydroponics until bud outgrowth was seen in UT plants (final age 50 days). Two biological RNA replicates were prepared from 5 plants for each treatment regime and from controls.

Dexamethasone based induction of RFLΔGR

15 day old T1 seedlings of *pUGNRFL:GR -2* and control wild type seedlings of similar age were treated with 10μM dexamethasone or with 0.1% ethanol as mock control. Five plants per treatment were grouped to form two biological replicates and the treatment regime was for 9h. RNA was prepared from culm tissues of these groups of plants (wild type and *pUGNRFL:GR -2*) by Trizol method (TRI reagent, Sigma) as per manufacturer's instructions.

PAT Assay

^{3}H-IAA (Amersham, TRK781) was a kind gift from Dr. Y Sreelakhsmi, University of Hyderabad, India. The polar auxin transport assay was adapted from Al-Hammadi *et al*., (2003). 1.2 cm stem section (nodal cutting) and 1.2cm root segments were pre incubated in 1uM IAA (Sigma) or 10uM NPA (Sigma) in ½ MS liquid media and kept on shaker to avoid gravitropic response. Segments were then placed on glass slides with their apical end (for basipetal transport) touching donor blocks (1.5% [w/v] agar in ½ MS containing 500nM of IAA and

500nM of ^{3}H-IAA , Amersham -TRK 781) and their basal ends touching receiver blocks (1.5% [w/v] agar in 1/2 MS). Each agar block was approximately 0.5cm X 0.5cm X 0.2cm in dimension. After 4 hours of incubation in a humidified chamber, under continuous light, the donor or receiver agar blocks were incubated in 1ml scintillation fluid for 1day and the radioactivity counts were measured in Beckman scintillation counter.

Supplemental References

Al-Hammadi A.S.A, Sreelakshmi Y, Negi S, Siddiqi I, and Sharma R. 2003. The *polycotyledon* Mutant of Tomato Shows Enhanced Polar Auxin Transport. *Plant Physiology*, 133, 113–125

Prasad K, Kushalappa K, Vijayraghavan U. 2003. Mechanism underlying regulated expression of *RFL*, a conserved transcription factor, in the developing rice inflorescence. *Mechanism of Development* **120,** 491–502.

Saghai-Maroof MA, Soliman KM, Jorgensen RA, Allard RW. 1984. Ribosomal DNA spacer-length polymorphisms in barley: mendelian inheritance, chromosomal location, and population dynamics. *Proceedings of National Academy of Sciences, USA* **81**, 8014-8018.

Schwab R, Ossowski S, Riester M, Weigel D. 2006. Highly specific gene silencing by artificial microRNAs in *Arabidopsis. The Plant Cell* **18**, 1121-1133.

Umehara M, Hanada A, Yoshida S, *et al.,* 2008. Inhibition of shoot branching by new terpenoid plant hormones. *Nature* **455**, 195 - 200

Supplemental Materials Legends

Fig. S1. Expression of hairpin loop RNA in *dsRNAiRFL* plants by RT-PCR analysis using leaf tissues. (A) Schematic of the T-DNA segment in *dsRNAiRFL* transgenics with the position of primers used for detecting expression of antisense transcripts. (B) RT-PCR for validating the expression of antisense strand in the transgenic hairpin RNA. Actin is used as control. Numbers

indicate the line number of plants. (C) Schematic of T-DNA segment in *amiR RFL* transgenic plants with the position of primers used for detecting expression of hygromycin transcripts in the leaf tissue that is shown in (D) with Actin as a control. Numbers indicate the line number. (E) Flowering time analysis for wild type (WT), *dsRNAiRFL* and *amiR RFL*. Delayed flowering in *dsRNAiRFL* plants (89 days) and *amiR RFL* plants (100 days) as compared to the control tissue culture regenerated WT plants (64 days). ***, $P<0.01$. the error bars indicate the standard error of the mean.

Fig. S2. Transcripts for *D3* and *RFL* in various rice tissues. Number in parenthesis refers to the PCR cycle number used for respective primer pair.

Fig. S3. Transgene copy number determination for *Ubi amiRD3*, *dsRNAiRFL* lines and *amiR D3-dsRNAiRFL* lines. Numbers on X-axis indicates the line number for the different genotypes. S1 = standard line with single T-DNA copy, S2 = standard line with two T-DNA copies and S3 = standard line with three T-DNA copies. Vertical bar represents the mean ΔCt value and the error bars indicate the standard error of the mean (n = 3). **, $P < 0.01$ gives the significance value for the Ct value of the single copy lines *Vs.* the standard with two copies of transgene.

Fig. S4. Plant height in *dsRNAiRFL* plant (B), *Ubi amiRD3* (C), *amiR D3-dsRNAiRFL* (D) and control wild type plant (A). (E) Comparative analysis of plant height measured after panicle emergence.

Fig. S5. Strigolactone analog (GR24) affects expression status of an auxin transporter gene *OsPIN1* in wild type plants. (A) *OsPIN1* transcript levels were determined in culm tissues of plants at three developmental growth stages. Treatment of 24h was with varied GR24 concentrations. RT-qPCR data for fold-change in expression of treated vs. untreated plants is plotted for the three different age groups. The error bars represent the standard error of the mean (n = 6). A fold-change of 1.5 was taken to categorize genes affected by GR24 treatment and is indicated by the dotted line in the graph. (B) Effect of prolonged 20 day GR24 treatment on *OsPIN1* transcript levels in the vegetative culm of plants. The plants were 35 or 50 days of age at

end of treatment and RT-qPCR data analysis was done as in panel A. (C) Representative pictures of 35 day old plants after long term treatment with varied concentrations of GR24. (D) Number of active buds per plant (n = 10) after 20 days of treatment given to plants of initial age 15 days (black bar) and 30 days (grey bar). * indicates significant (*P<0.01*) reduction in active bud number per plant after 20 days of GR24 treatment to plants of initial age 30 days as compared to UT. UT = control treatment with acetone, in all cases. Effect of GR24 on the transcript levels of *OsPIN3* in the vegetative culm harvested at at three developmental growth stages after 24hrs treatment (E) and two developmental stages after 20 day treatment (F).

Fig. S6. Live imaging of auxin responsive promoter driven GFP reporter activity in young *dsRNAiRFL* and wild type plantlets (WT). (A) Schematic of the T-DNA segment of *IR4DR5:GFP* construct. (B) Schematic of the T-DNA segment of *dsRNAiRFL-IR4DR5:GFP* construct. (C) WT control showing autoflorescence of chlorophyll (red) upon excitation at 480nm. (D) *IR4DR5:GFP* shows high activity at the basal region of a young seedling indicating functional polar auxin transport. (E) *dsRNAiRFL-IR4DR5:GFP* seedling manifests a diffused auxin pattern throughout the seedling suggestive of altered auxin transport. Immuno histochemistry analysis for OsPIN2/OsPIN3 in histological sections of WT and *amiR RFL* tissues. (F) Western Blot analysis to show cross-reactivity of Anti-AtPIN2 antisera to *in vitro* translated OsPIN3 and AtPIN2 proteins. Immunolocalization of OsPIN3 in vegetative SAM of WT (I) and *amiR-RFL* plants (J). Delayed vasculature development is observed in axillary buds of *amiR-RFL* (G) as compared to that of WT (H, yellow arrows). (K) Fluorescence intensity measured in the vegetative shoot apices of WT and *amiR-RFL* (I and J) Scale bar = 50μm.

Supplementary Table S1. Primers used in this study

	Forward primer (5'-3')	Reverse primer (5'-3')	Use
Actin	CTACGAGCTTCCTGATGGAC	GATGGATCCTCCAATCCAGAC	qRT-PCR
RFLE2	GTTGTCCGAGGAGCATGAC	CCTCGCCGGGCTCCGTCACC	Genotyping
LAX1	ATGCTGGAGCAGGCCATCCA	GTTCAGCTCAAGGGCCAGA	qRT-PCR
D3	GGTATCGAATCACTGCAGAC	CTCTGGGGCTGGATAATAGT	qRT-PCR
D10	CTATTGTAAGCTCCGACGAT	CTAGTCTTCTCGGCTACAGAT	qRT-PCR
HTD1	TGGCTATGTTCTTCTTGTAGAG	AGTTAGACTGGATCTGATGCTT	qRT-PCR
MOC1	TTCTGCTTGTTGCCTTCC	ATCATTACCCACCAAAAG	qRT-PCR
CUC1	GACTTCTGGAACTAATCC	AAGGAGGAAGAGAAGGAT	qRT-PCR
OsTB1	GCCGGATGCAAGAAATC	TCAGCAGTAGTGCCGCGAA	qRT-PCR
OsPIN1	AGTACAAAGCTTGGGGGGAC	ATCTCTTGTCAGAATCGGCG	qRT-PCR
OsPIN3	ATCCTGAGCACAGCGGTAAT	CAATGTCCGACAACAGGCTA	qRT-PCR
CUC3	TTTATGAGATGGGCCCACCT	GCA AGC TAG GTA CAC ACC TG	qRT-PCR
OSH1	CCTGAAGCAGATCAACAACT	CGATCTAGGTCATGGTAGCTG G	qRT-PCR
I miRD3-s	AGTAACATGCCCGATAATGACAT CAGGAGATTCAGTTTGA		Cloning
II miRD3-a	AATGATGTCATTATCGGGCATGTT ACTGCTGCTGCTACAGCC		Cloning
III miRD3*s	CTATGTCTTTAACGGGCATGTTAT TCCTGCTGCTAGGCTG		Cloning
IV miRD3*a	AATAACATGCCCGTTAAAGACAT AGAGAGGCAAAAGTGAA		Cloning
T7	CGGGATATCACTCAGCATAATG		Cloning
RFLEAR		TTAAGCGAAACCCAAACGGAG TTCTAGATCCAGATCTAG	Cloning

I2B	AGCTTTGTTTAAACCTGCAGGTGC AATCCA	GCGAGGATGGCTACTAGCCTA GAGC	Cloning
I miR RFL-s	AGTTTAGAACGACCTAACTGCCT CAGGAGATTCAGTTTGA		Cloning
II miR RFL-a	TGAGGCAGTTAGGTCGTTCTAAA CTGCTGCTGCTACAGCC		Cloning
III miR RFL*s	CTAGGCAGTTAGGTCGTTCTAAAT TCCTGCTGCTAGGCTG		Cloning
IV miR RFL*a	AATTTAGAACGACCTAACTGCCT AGAGAGGCAAAAGTGAA		Cloning
OsIAA7	TAATCCATGCAGCTAATAACCTCT	GTTGGTTAAGTAGGAACAGGA AAACC	qRT-PCR
OsIAA20	TTGTACGTGAACGGGATTATTTG	CATGCTTATGAAATTGCTGAA ACA	qRT-PCR
OsKNAT1	TTT CAC CGT GGA TTG ACA AG	AGATAGGCAACTTTGAACACC	qRT-PCR
AtPIN2 T7	CCTAATACGACTCACTATAGGGA GCCACC ATG GTT GCG GTT TTC GCG G	TAA CCA AGC AAG GCC AAA GAG ACT	PCR for *in vitro* transcription
OsPIN3 T7	CCTAATACGACTCACTATAGGGA GCCACCATG GAC ACG CTG CAG AAG CTG	TAA TGCGTCCGAGAGAATGGAGAT	PCR for *in vitro* transcription

Supplementary Table S2: Polar auxin transport assay in seed grown ~90 day old shoot and root tissues. Amount of ^{3}H-IAA transported in basipetal direction was measured in wild type stem and root (pre-treated with 1uM IAA or 10uM NPA) and *amiR-RFL* stems and roots pretreated with 1uM IAA .

Supplementary Table S2

^{3}H IAA in Stem

Treatment and Plant	Cpm	
**Basipetal (pre incubated in 1um IAA)**		
	Donor	Receiver
WT	68,346	648
amiR RFL	57,532	336
amiR RFL	65,164	419
**Basipetal (pre incubated in 10uM NPA)**		
WT	1,02,103	359

^{3}H IAA in Roots

Treatment and Plant	Cpm (Sum total of 3 roots per plant)	
**Basipetal (pre incubated in 1um IAA)**		
	Donor	Receiver
WT	1,36,439	1920
amiR RFL	1,56,553	519
amiR RFL	1,50,796	682
**Basipetal (pre incubated in 10uM NPA)**		
WT	94,762	397

Supplementary Figure S1

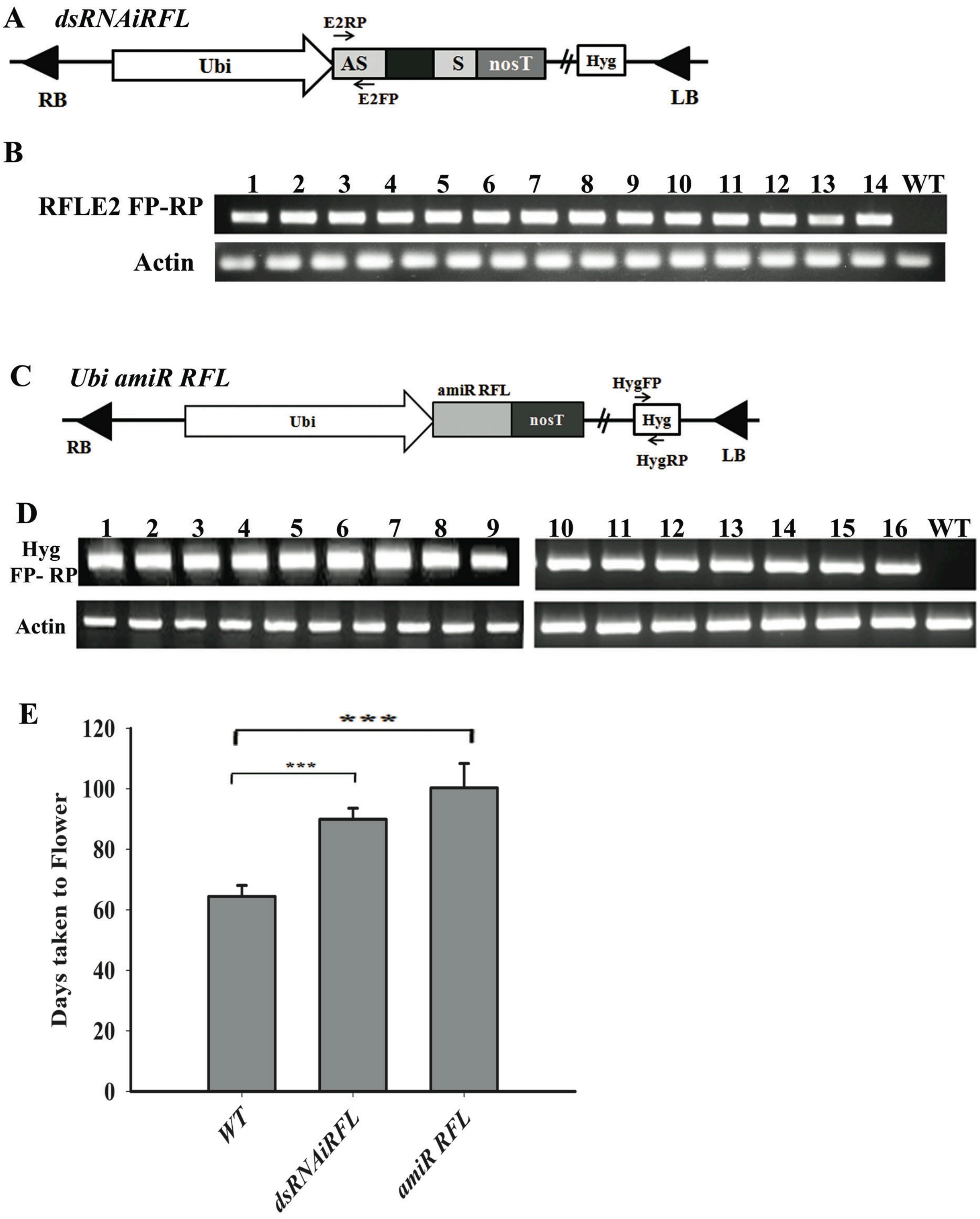

Supplementary Figure S2

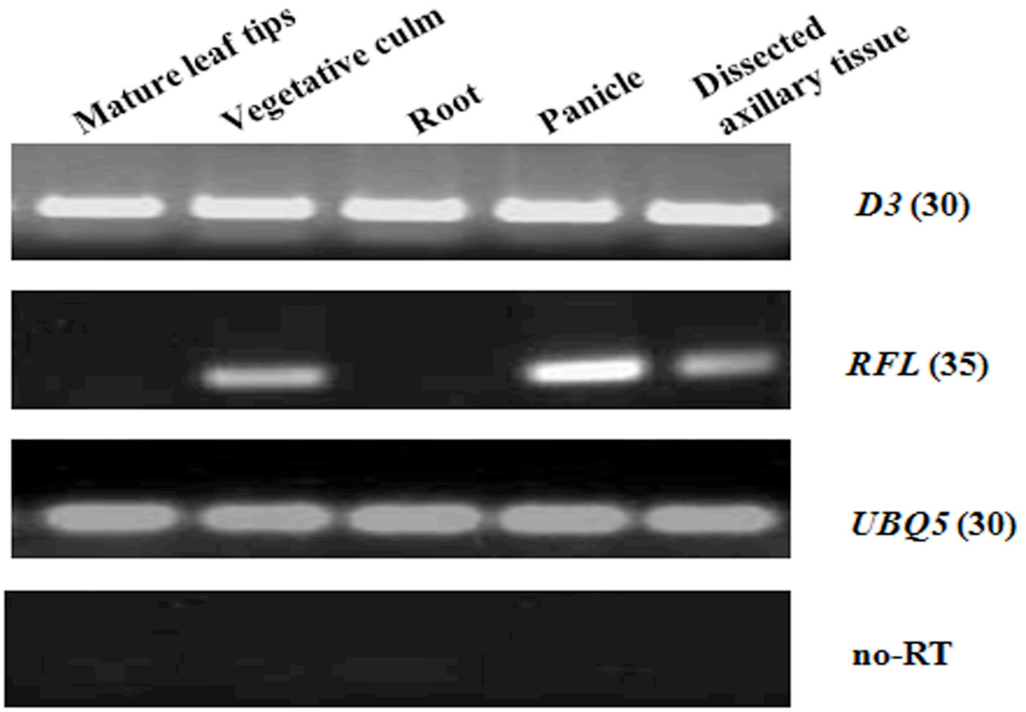

Supplementary Figure S3

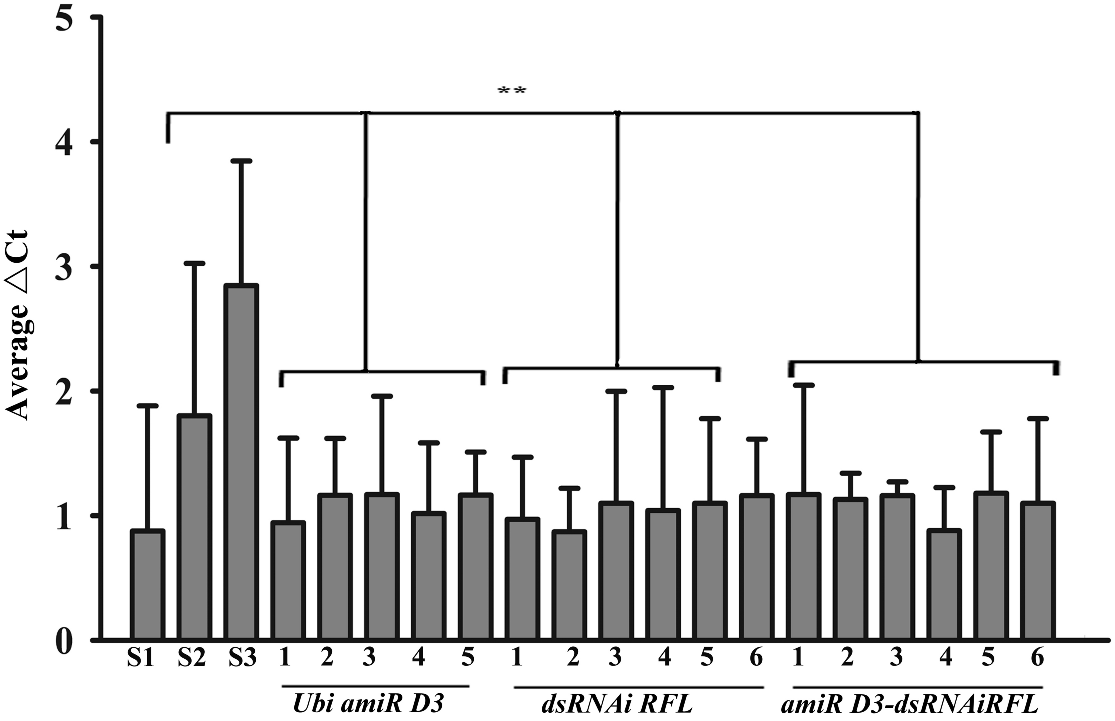

Supplementary Figure S4

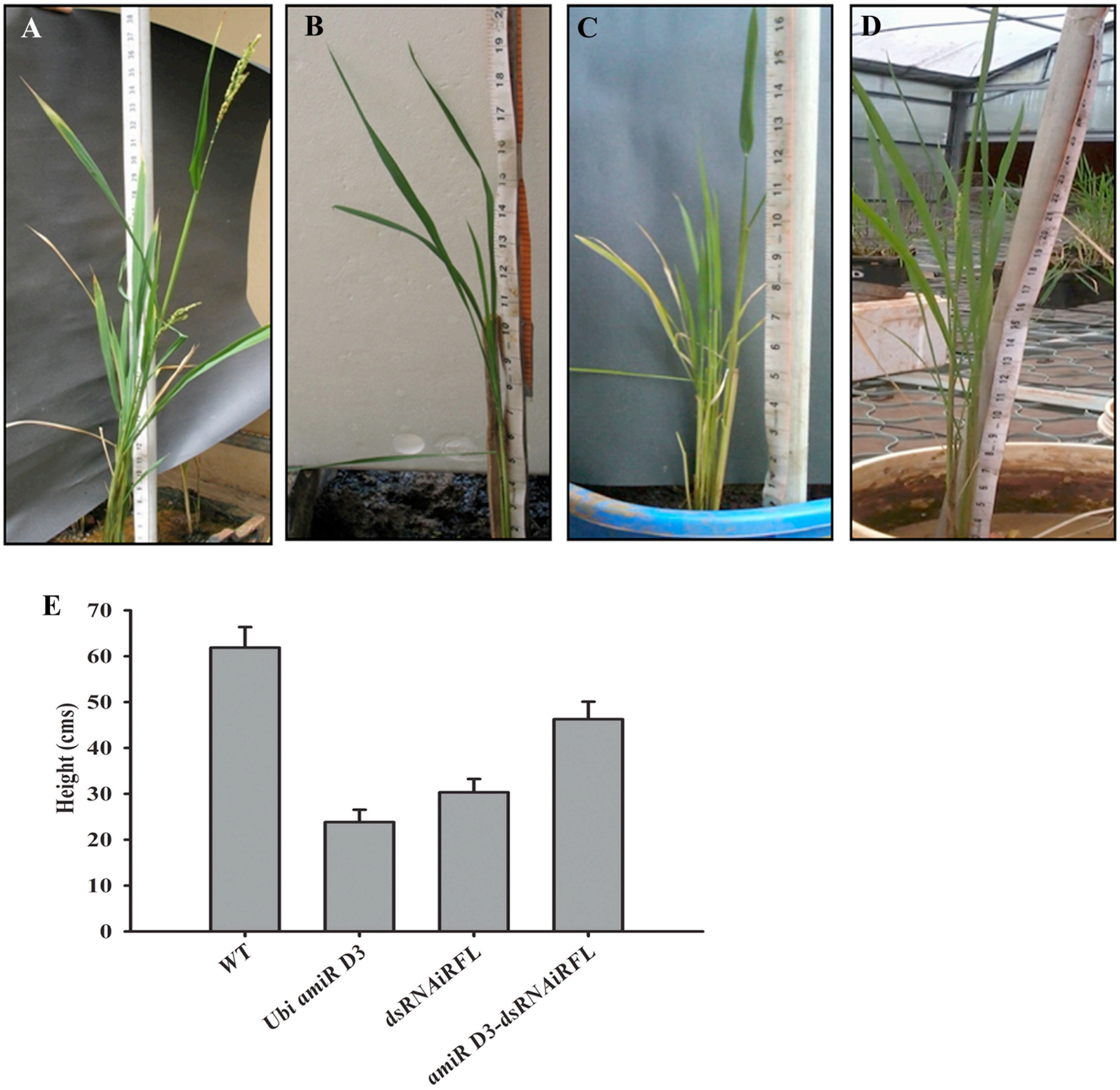

Supplementary Figure S5

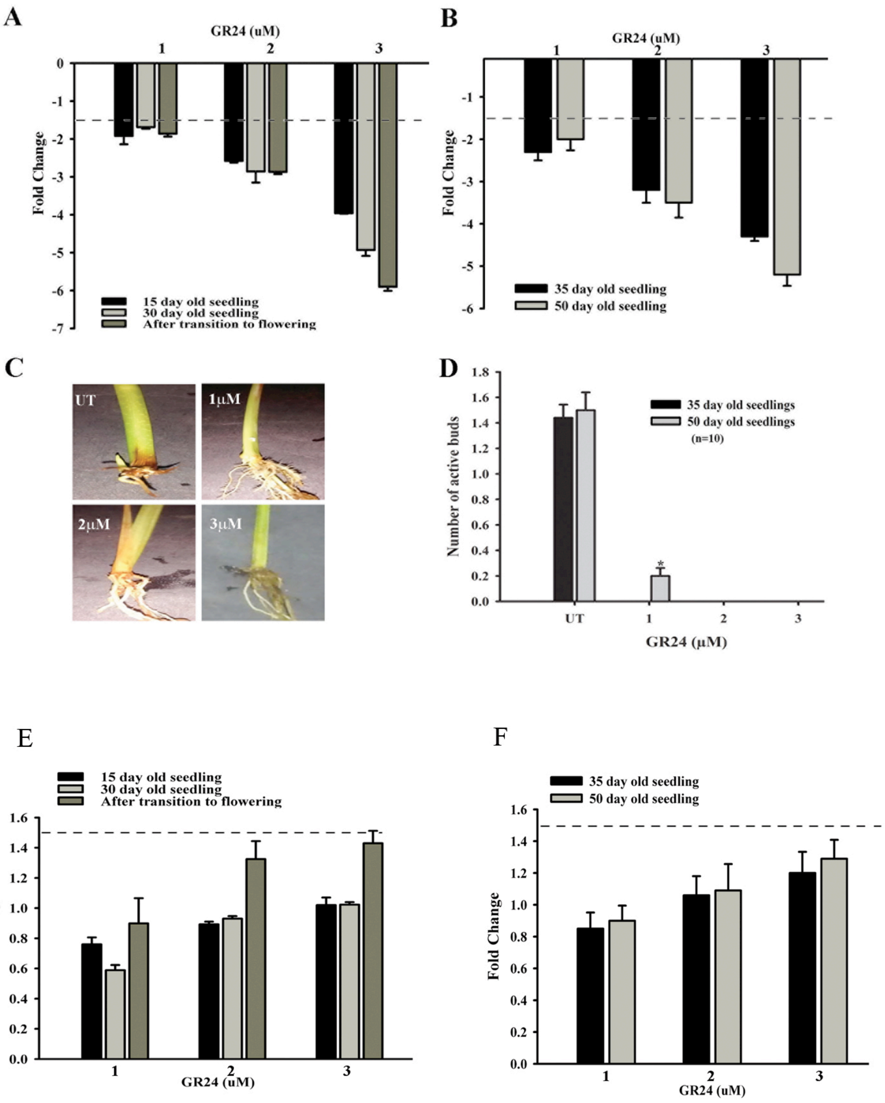

Supplementary Figure S6.

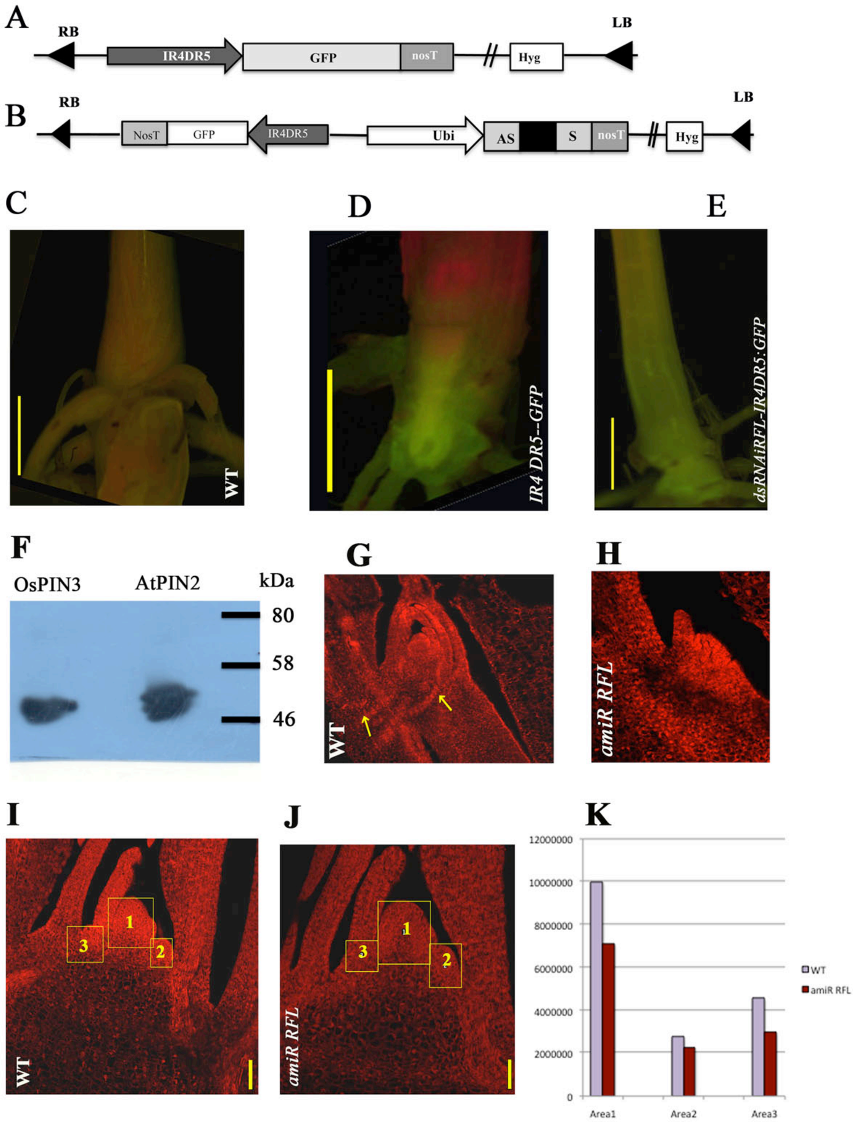

Supplement: Supplementary Data [file supp_erv092_jexbot135392_file001.pdf]
